# Synthesis of New Imidazopyridine Nucleoside Derivatives Designed as Maribavir Analogues

**DOI:** 10.3390/molecules25194531

**Published:** 2020-10-03

**Authors:** Georgios Papadakis, Maria Gerasi, Robert Snoeck, Panagiotis Marakos, Graciela Andrei, Nikolaos Lougiakis, Nicole Pouli

**Affiliations:** 1 Division of Pharmaceutical Chemistry, Department of Pharmacy, National and Kapodistrian University of Athens, Panepistimiopolis-Zografou, 15771 Athens, Greece; gpapadakis@pharm.uoa.gr (G.P.); mairager@pharm.uoa.gr (M.G.); marakos@pharm.uoa.gr (P.M.); pouli@pharm.uoa.gr (N.P.); 2 Laboratory of Virology & Chemotherapy, Rega Institute, KU Leuven, 3000 Leuven, Belgium; robert.snoeck@kuleuven.be (R.S.); graciela.andrei@kuleuven.be (G.A.)

**Keywords:** benzimidazole, nucleosides, imidazo[4,5-b]pyridine, HCMV, antiviral activity, antiproliferative activity

## Abstract

The strong inhibition of Human Cytomegalovirus (HCMV) replication by benzimidazole nucleosides, like Triciribine and Maribavir, has prompted us to expand the structure–activity relationships of the benzimidazole series, using as a central core the imidazo[4,5-b]pyridine scaffold. We have thus synthesized a number of novel amino substituted imidazopyridine nucleoside derivatives, which can be considered as 4-(or 7)-aza-d-isosters of Maribavir and have evaluated their potential antiviral activity. The target compounds were synthesized upon glycosylation of suitably substituted 2-aminoimidazopyridines, which were prepared in six steps starting from 2-amino-6-chloropyridine. Even if the new compounds possessed only a slight structural modification when compared to the original drug, they were not endowed with interesting antiviral activity. Even so, three derivatives showed promising cytotoxic potential.

## 1. Introduction

Human cytomegalovirus (HCMV) is a prevalent herpesvirus, with IgG antibodies indicating past infection found in approximately 60% of adults in developed countries and almost 100% in developing ones [1]. Although HCMV infection rarely leads to clinical manifestations in immunocompetent hosts, there is an increasing amount of data associating lifelong viral persistence with vascular diseases (atherosclerosis [2], hypertension [3]) and the progression of some cancer types [4]. In addition, HCMV is a major opportunistic pathogen in immunocompromised individuals, posing a serious threat to neonates, allograft recipients and AIDS patients [5]. Perinatal infection can cause irreversible hearing loss, blindness and mental retardation, while immunosuppressed adults may develop multi-organ failure syndrome, which is a life-threatening condition [1,5].

Ganciclovir (GCV) and its orally bioavailable prodrug, Valganciclovir, have served as gold standards for pre-emptive therapy and prophylaxis against HCMV in solid organ transplant patients [6] as well as for the treatment of CMV retinitis [7] for almost 30 years. However, their myelosuppressive potential precludes their prophylactic use in stem cell transplant recipients, which has led to their administration, mostly upon engraftment [8]. In addition, mutations mapping in the gene encoding for the UL97 kinase (responsible for the first phosphorylation of GCV towards its active triphosphate form) and in the UL54 gene encoding for the DNA polymerase (target of ganciclovir triphosphate), have led to the emergence of drug resistant strains [9]. Furthermore, the DNA polymerase inhibitors Cidofovir and Foscarnet, which are currently approved as second line agents, have several drawbacks that limit their clinical use, namely severe side effects and poor pharmacokinetic properties [10].

**Figure 1:**
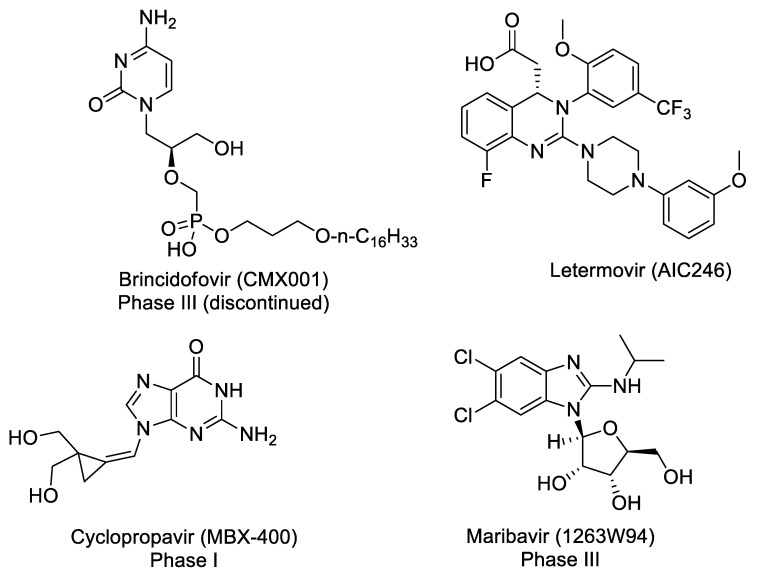
Recently developed antiviral agents against HCMV.

It has become clear that there is an urgent need for better tolerated and more effective antiviral drugs, in order to fully address the health risks posed by HCMV. There are four compounds that have been considered or are at an advanced stage of clinical development for this purpose (Figure 1). Brincidofovir is a per os administered hexadecyloxypropylester of Cidofovir aimed at addressing the parent compound’s dose-limiting renal toxicity [11]. The development of Brincidofovir for therapy of HCMV has been halted because of increased gastrointestinal toxicity of the oral formulation in adult hematopoietic cell transplant recipients. The non-nucleoside guanosine analogue Cyclopropavir, which shares the same mechanism of action with GCV, has proven to be more potent in HCMV inhibition in vitro [12] and Phase I trials have been recently completed. Furthermore, the search for novel molecular targets within the viral life cycle has led to the fast track approval of the terminase inhibitor Letermovir in late 2017, for the prophylaxis of HCMV infection and disease in adult HCMV-seropositive recipients of an allogeneic human stem cell transplant (HSCT) [13]. Phase II clinical trials are also about to be launched for the use of Letermovir in paediatric patients who underwent an HSCT.

At the same time, the benzimidazole l-riboside Maribavir is about to be evaluated in Phase III trials involving transplant recipients with HCMV infections that are refractory or resistant to the currently approved drugs as well as for its potential superiority over Valganciclovir in HSCT patients. Maribavir was developed in the late 1990s and has been proven to inhibit viral DNA synthesis as well as nucleocapsid egress from the nucleus via the inhibition of the viral kinase UL97 [14]. However, initial Phase III clinical trials failed to prove sufficient benefits for post-transplant patients. This has later on been accredited to the pre-emptive therapy with GCV that several patients had received prior to surgery and to the low daily administered dose [15].

As a continuation of our previous involvement with the synthesis and evaluation of purine isosteric bioactive nucleosides [16,17,18,19], we designed and synthesized a number of novel nucleoside derivatives, which can be considered as 4-(or 7)-aza-d-isosters of Maribavir, having in mind that the d-enantiomer of Maribavir possesses interesting anti-HCMV properties as well [20]. Our goal was to expand the structure–activity relationships of the benzimidazole series to the less-studied and more “purine-like” imidazo[4,5-*b*]pyridine scaffold, retaining the pattern of the 5,6-dichlorosubstitution in the new compounds. The diverse nature of the 2-amino groups in each of the final pair of isomeric nucleosides was at the core of our attempt to explore the spatial limitations of possible target enzymes as well as to gain some insight on potential interactions developed. Within this context, we disclose herein the preparation and pharmacological evaluation of the 1- and 3-regioisomeric *β*-d-ribosides of 5,6-dichloroimidazo[4,5-*b*]pyridine, introducing various amino substituents at the vacant position of the imidazole ring.

## 2. Results and Discussion

### 2.1. Chemistry

**Scheme 1:**
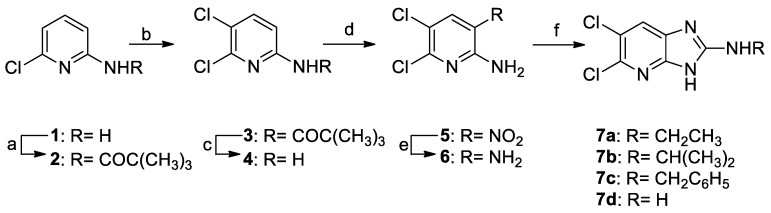
Reagents and conditions: (**a**) pivaloyl chloride, Et_3_N, DCM, r.t., 22 h; (**b**) NCS, DMF, 100 °C, 18 h; (**c**) conc. HCl, H_2_O, EtOH, reflux, 2 h; (**d**) conc. HNO_3_, conc. H_2_SO_4_, r.t., 45 min; (**e**) SnCl_2_·2H_2_O, conc. HCl, 60 °C, 40 min; (**f**) (i) *N*-alkylisothiocyanate, dry THF, reflux, 36 h, (ii) HgO, dry THF, reflux, 40 min for **7a**–**c**, or BrCN, MeOH, H_2_O, 24 h for **7d**.

The synthetic route we envisaged in order to gain access to the target nucleosides involved the direct glycosylation of the suitably substituted imidazo[4,5-*b*]pyridines **7a**–**d** (Scheme 1). A common intermediate for the synthesis of these derivatives is 5,6-dichloropyridine-2,3-diamine (**6**), which was prepared following a five-step procedure, previously described by our group, starting from the pyridinamine **1** [21].

Amino substituted imidazopyridine derivatives **7a**–**c** were prepared using a one-pot-two-stage procedure, which involves an initial treatment of **6** with appropriately substituted *N*-alkylisothiocyanates in refluxing THF. The resulting mixture of isomeric thioureas undergoes rapid cyclodesulfurization in the presence of mercury oxide [22], yielding compounds **7a**–**c**. Concerning the preparation of the primary aminoderivative **7d**, we implemented a different approach, which had been previously reported by Townsend [23], for the synthesis of the corresponding benzimidazole analogue. Thus, the addition of cyanogen bromide in a suspension of diamine **6** in a 1:1 mixture of MeOH and water provided **7d** as the sole reaction product in very good yield.

**Scheme 2:**
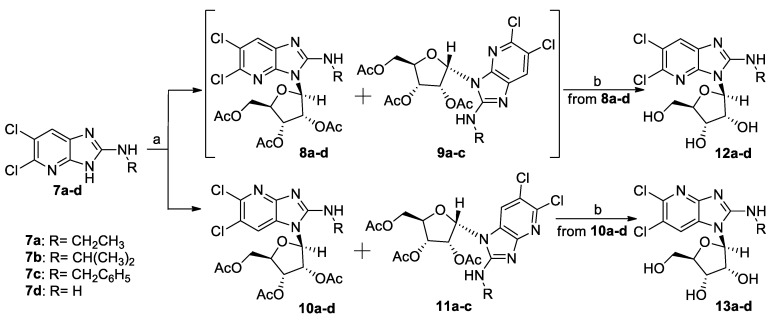
Reagents and conditions: (**a**) (i) *N*,*O*-bis(trimethylsilyl)acetamide, ACN, reflux, 2h, (ii) tetra-*O*-acetyl-*β*-d-ribofuranose, TMSOTf, reflux, 3h; (**b**) NH_3(g)_, MeOH, rt, 18h.

The target *N*1- and *N*3-ribonucleoside acetates were prepared under modified Vorbrüggen conditions. The in situ formation of the persilylated heterocyclic bases by treatment of the corresponding heterocyclic compounds **7a**–**d** with *N*,*O*-bistrimethylsilylacetamide (BSA) was followed by the addition of peracetylated *β*-d-ribofuranose and trimethylsilyltrifluoromethanesulfonate (TMSOTf) (Scheme 2). Thus, concerning the glycosylation reaction of compounds **7a**–**c**, the major products isolated were the 3-*β*-d-ribosides **8a**–**c**, as a mixture with a small amount of the corresponding *α*-anomers **9a**–**c**. The *N*1 regioisomers were also obtained as mixtures of the 1-*β*-d ribosides **10a**–**c** with their corresponding *α*-d anomers **11a**–**c**.

Regarding the *N*1 regioisomers, we were able to isolate pure *N*1-*β*-d nucleoside acetates **10a**–**c** as well as their corresponding *N*1-*α*-d anomers **11a**–**c**, upon chromatographic purification. The site of ribosylation as well as the anomeric configuration were unambiguously determined on the basis of NOE spectroscopy. Taking into consideration the NOE spectra of compounds **10a**–**c** and **11a**–**c**, we observed clear correlation peaks between the aromatic proton and protons of the furanose ring, determinant of the *N*1-ribosylation pattern. In addition, we also noticed cross correlation peaks between 1′-H and 4′-H of the sugar moiety in the spectra of compounds **10a**–**c**. Such peaks were not observed on the NOE spectra of **11a**–**c**, thus clearly concluding that **11a**–**c** were the *N*1-*α*-d nucleoside products of the reaction, while **10a**–**c** were their corresponding *β*-d anomers. Deacetylation of **10a**–**c** with methanolic ammonia provided the final compounds **13a**–**c**.

Isolation of the pure 3-*β*-d nucleoside acetates proved to be difficult at this stage, so the mixtures of **8a**–**c** with their corresponding *α*-anomers **9a**–**c** were subjected to ammoniolysis, to provide the deprotected nucleosides. The ethylamino (**12a**) and isopropylamino (**12b**) derivatives were isolated in pure form by recrystallization, whereas an analytically pure sample of the benzylamino compound **12c** was obtained upon purification with semi-preparative HPLC. Anomeric purity and configuration of compounds **12a**–**c** were determined on the basis of ^1^H-NMR and NOE spectra, respectively. In the latter ones, we observed clear correlation peaks between 1′-H and 4′-H of the ribofuranose moiety, while there was a profound absence of correlation peaks between the aromatic and sugar protons on the NOE spectra of each of the aforementioned compounds. The close examination of 1D and 2D NMR spectra reveals that a simple differentiation between each pair of regio isomers can be easily made upon inspection of the chemical shift of the aromatic proton. This proton appears upfield in the case of the N3-isomers **12a**–**d** (7.7–7.8 ppm) whereas it shifts downfield (8.0–8.1 ppm) concerning the N1-isomers **13a**–**d**.

**Figure 2:**
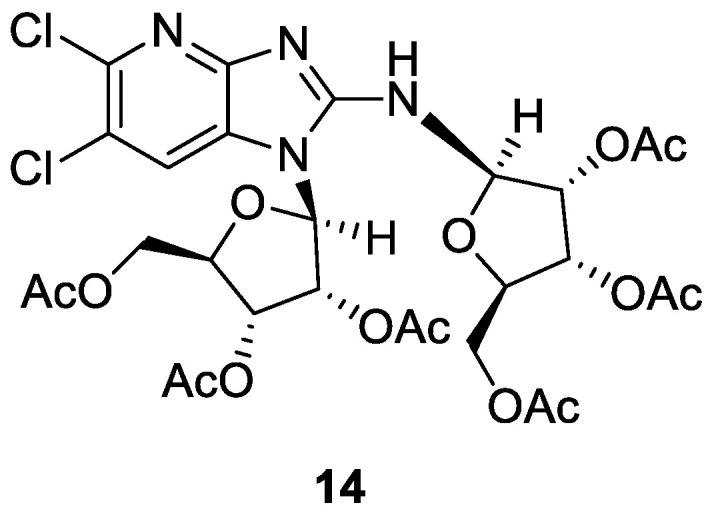
Structure of di-ribosylated by-product formed upon glycosylation of the amino derivative **7d**.

Applying the same reaction conditions for the glycosylation of the primary aminoderivative **7d**, we isolated from the reaction mixture the 3-*β*-d nucleoside acetate (**8d**), as well as the 1-*β*-d isomer (**10d**), without detectable amounts of the corresponding *α*-anomers. Deprotection of **8d** and **10d** with methanolic ammonia provided the respective final ribosides **12d** and **13d**. However, the major product of the glycosylation reaction of **7d** turned out to be the di-ribosylated compound **14** (Figure 2), whose structure was elucidated on the basis of ^1^H-NMR, ^13^C-NMR, NOE and mass spectra. Two sets of sugar carbon peaks were observed on the ^13^C-NMR spectrum of **14**, in the presence of only one set of aromatic carbon peaks. The sites of substitution as well as the anomeric configuration were assigned as exemplified in Figure 2 (namely *N*,1-*β*-d). Upon careful examination of the NOE spectrum of **14**, we observed a set of correlation peaks between the aromatic proton and protons of one sugar moiety as well as correlation peaks between the anomeric proton of each furanose with its corresponding 4′-proton.

### 2.2. Biological Evaluation

Compounds **12a**–**d** and **13a**–**d** were evaluated for their activity against Vaccinia virus, Adeno virus-2, Human Coronavirus (229E), HSV-1, HSV-2, VZV, and HCMV (AD-169 and Davis strains) in human embryonic lung cell cultures (HEL) (Tables S1–S3). Unfortunately, the new derivatives proved to lack antiviral activity against all viruses tested. In particular, the substitution of the benzimidazole pharmacophore present in Maribavir by the imidazo[4,5-*b*]pyridine scaffold, in combination with the replacement of the l-sugar configuration with its corresponding d-riboside, resulted in total loss of the anti-HCMV activity. Nevertheless, upon determination of the cytotoxic properties of the new derivatives, compounds **12c**, **12d** and **13c**, proved to possess moderate antiproliferative activity against the three cancer cell lines tested, namely human T-lymphocyte cells (CEM), human cervix carcinoma cells (HeLa) and human dermal microvascular endothelial cells (HMEC-1) (Table S4). Among these compounds, both 2-benzylamino-substituted derivatives **12c** and **13c** showed antiproliferative activity against CEM cell-line, with equipotent IC_50_ values (37 ± 3 and 39 ± 8µM) and at the same time, **12c** proved active in inhibiting the growth of HeLa and HMEC-1 cell lines, possessing IC_50_ values of 36 ± 7 µM and 20 ± 2 µM, respectively. The increased antiproliferative effects of **12c** and **13c** are worth investigating further and are currently under active search in our laboratories.

## 3. Materials and Methods

### 3.1. General Information

Tetrahydrofuran (THF) was distilled from sodium/benzophenone ketyl immediately prior to use. Melting points were determined on a Büchi apparatus and are uncorrected. ^1^H-NMR spectra and ^2^D (COSY, NOESY, HSQC, HMBC) NMR spectra were recorded on a Bruker Avance III 600 or a Bruker Avance (Bruker, Karlsruhe, Germany) DRX 400 instrument, whereas ^13^C-NMR spectra were recorded on a Bruker Avance III 600 in deuterated solvents and were referenced to TMS (*δ* scale). The signals of ^1^H and ^13^C spectra were unambiguously assigned by using 2D NMR techniques: ^1^H^1^H COSY, NOESY, HSQC and HMBC. Mass spectra were recorded with a LTQ Orbitrap Discovery instrument, possessing an Ionmax ionization source. Column chromatography was performed on Merck (Merk, Darmstadt, Germany) silica gel 60 (0.040–0.063 mm), unless specified otherwise. Analytical thin layer chromatography (TLC) was carried out on precoated (0.25 mm) Merck silica gel F-254 plates. Preparative HPLC was performed on a system equipped with two Prep LabAlliance pumps (ASI, Richmond, CA, USA), a Fortis C-18 (5μm) column (i.d. 10 × 250 mm) and a FLASH 06S DAD 600 detector (ECOM, Praha, Czech Republic). Optical rotations were obtained on a Perkin-Elmer 341 Polarimeter (Perkin Elmer, Shelton, CT, USA).

### 3.2. Synthesis

5,6-Dichloro-*N*-ethyl-3*H*-imidazo[4,5-b]pyridin-2-amine(**7a**): Ethylisothiocyanate (1 mL, 11.24 mmol) was added to a solution of diamine **6** (500 mg, 2.81 mmol) in anhydrous THF (10 mL) and the reaction mixture was refluxed under argon for 36 h. The mixture was then cooled to room temperature and HgO (1.22 g, 5.65 mmol) was added, leading to an orange suspension. Refluxing the reaction mixture resulted progressively in a black coloured suspension and within 40 min the reaction was completed. The mixture was filtered warm through a celite pad, which was thoroughly washed with warm MeOH. The solvent was then vacuum-evaporated and the residue was purified by column chromatography using a mixture of CHCl_3_/MeOH (95/5 to 85/15, *v*/*v*) as the eluent, to result in 340 mg of **7a** (52% overall yield). Beige solid, mp > 300 °C _(dec)_, (MeOH). ^1^H-NMR (DMSO-*d*_6_, 600MHz) *δ* 1.16 (t, 3H, CH_3_, *J* = 7.2 Hz), 3.33 (m, 2H, CH_2_), 7.37 (br s, 1H, D_2_O exchangeable, NH), 7.57 (s, 1H, H-7). ^13^C-NMR (151 MHz, DMSO-*d*_6_) *δ* 14.87 (CH_3_), 36.82 (CH_2_), 116.40 (C-6), 116.58 (C-7, major tautomeric form), 121.73 (C-7, minor tautomeric form), 126.92 (C-7a), 137.46 (C-5), 156.20 (C-3a), 158.92 (C-2). HR-MS (ESI) *m*/*z*: Calcd for C_8_H_9_Cl_2_N_4_: [M + H]^+^ = 231.0199, found 231.0202.

5,6-Dichloro-*N*-isopropyl-3*H*-imidazo[4,5-b]pyridin-2-amine (**7b**):This compound was prepared by a procedure analogous to that described for **7a**, upon reaction of diamine **6** (500 mg, 2.81 mmol) with isopropyl isothiocyanate (0.90 mL, 8.43 mmol). Purification was effected by column chromatography, using a mixture of DCM/EtOAc (70/30 to 20/80, *v*/*v*) and then EtOAc/MeOH (99/1 to 97/3, *v*/*v*) as eluents. Overall yield 55%. White solid, mp 235–236 °C (acetone). ^1^H-NMR (600 MHz, DMSO-*d*_6_) *δ* 1.20 (d, 6H, 2xCH_3_, *J* = 6.1 Hz), 3.92 (m, 1H, CH), 7.34 (br s, 1H, D_2_O exchangeable, NH), 7.56 (s, 1H, H-7), 11.06 (br s, 1H, D_2_O exchangeable, imidazole NH of the major tautomeric form), 11.66 (br s, 1H, D_2_O exchangeable, imidazole NH of the minor tautomeric form). ^13^C-NMR (151 MHz, DMSO-*d*_6_) *δ* 22.58 (CH_3_), 43.97 (CH), 116.37 (C-6), 116.61 (C-7 of the major tautomeric form), 121.62 (C-7 of the minor tautomeric form), 126.86 (C-7a), 137.41 (C-5), 156.22 (C-3a), 158.24 (C-2). HR-MS (ESI) *m*/*z*: Calcd for C_9_H_11_Cl_2_N_4_: [M + H]^+^ = 245.0355, found 245.0352.

*N*-Benzyl-5,6-dichloro-3*H*-imidazo[4,5-b]pyridin-2-amine (**7c**): This compound was prepared by a procedure analogous to that described for **7a**, upon reaction of diamine **6** (500 mg, 2.81 mmol) with benzyl isothiocyanate (0.80 mL, 6.03 mmol). Purification was effected by column chromatography, using a mixture of DCM/EtOAc (90/10 to 50/50, *v*/*v*) and then EtOAc/MeOH (99/1 to 90/10, *v*/*v*) as eluents. Overall yield 51%. White solid, mp: 277–278 °C (MeOH). ^1^H-NMR (600 MHz, DMSO-*d*_6_) *δ* 4.55 (d, 2H, CH_2_, *J* = 6.0 Hz), 7.24 (t, 1H, phenyl H-4′’, *J* = 7.2 Hz), 7.32 (t, 2H, phenyl H-3′’, H-5′’, *J* = 7.6 Hz), 7.36 (d, 2H, phenyl H-2′’, H-6′’, *J* = 7.3 Hz), 7.60 (s, 1H, H-7), 7.94 (br s, 1H, D_2_O exchangeable, NH), 11.38 (br s, 1H, D_2_O exchangeable, imidazole NH). ^13^C-NMR (151 MHz, DMSO-*d*_6_) *δ* 45.36 (CH_2_), 116.89 (C-6), 117.05 (C-7), 126.97 (phenyl C-4′’), 127.13 (C-7a), 127.25 (phenyl C-3′’, C-5′’), 128.33 (phenyl C-2′’, C-6′’), 137.50 (C-5), 139.34 (phenyl C-1′’), 155.88 (C-3a), 158.81 (C-2). HR-MS (ESI) *m*/*z*: Calcd for C_13_H_11_Cl_2_N_4_: [M + H]^+^ = 293.0355, found 293.0358.

5,6-Dichloro-3*H*-imidazo[4,5-b]pyridin-2-amine (**7d**): To a suspension of the diamine **6** (570 mg, 3.20 mmol) in MeOH (12 mL) and H_2_O (12 mL) was added BrCN (1.60 mL, 5M solution in acetonitrile, 8 mmol) and the reaction mixture was stirred at room temperature for 48 h. MeOH was concentrated under vacuo and the pH of the resulting aqueous phase was adjusted to 8 with a saturated NaHCO_3_ solution. The aqueous layer (150 mL) was extracted with EtOAc (5 × 200 mL) and the combined organic layers were dried (anhydrous Na_2_SO_4_) and concentrated to dryness. The residue thus obtained was triturated with diethyl ether to afford 510mg of **7d** as an amorphous solid (79%). ^1^H-NMR (DMSO-*d*_6_, 600MHz) *δ*7.05 (br s, 2H, D_2_O exchangeable, NH_2_), 7.58 (s, 1H, H-7). ^13^C-NMR (151 MHz, DMSO-*d*_6_) *δ* 117.54 (C-6), 118.24 (C-7), 129.57 (C-7a), 136.73 (C-5), 153.60 (C-3a), 158.85 (C-2). HR-MS (ESI) *m*/*z*: Calcd for C_6_H_5_Cl_2_N_4_: [M + H]^+^ = 202.9886, found 202.9887.

5,6-Dichloro-*N*-ethyl-3-(2′,3′,5′-tri-*O*-acetyl-*β*-d-ribofuranosyl)-3*H*-imidazo[4,5-b]pyridin-2-amine (**8a**), 5,6-dichloro-*N*-ethyl-3-(2′,3′,5′-tri-*O*-acetyl-*α*-d-ribofuranosyl)-3*H*-imidazo[4,5-b]pyridin-2-amine (**9a**), 5,6-dichloro-*N*-ethyl-1-(2′,3′,5′-tri-*O*-acetyl-*β*-d-ribofuranosyl)-1*H*-imidazo[4,5-b]pyridin-2-amine (**10a**) and 5,6-dichloro-*N*-ethyl-1-(2′,3′,5′-tri-*O*-acetyl-*α*-d-ribofuranosyl)-1*H*-imidazo[4,5-b]pyridin-2-amine (**11a**): To a suspension of the imidazopyridine **7a** (250 mg, 1.08 mmol) in dry CH_3_CN (10 mL) was added *N*,*O*-bis-(trimethylsilyl)acetamide (291 mg, 1.43 mmol) and the reaction mixture was refluxed for 2 h. The suspension was then cooled to room temperature and 1,2,3,5-tetra-*O*-acetyl-*β*-d-ribofuranose (413 mg, 1.30 mmol) was added, followed by the dropwise addition of trimethylsilyltriflate (0.3 mL, 1.54 mmol) at 0 °C. The mixture was refluxed for 3 h, the solvent was vacuum evaporated, the residue was dissolved in EtOAc (100 mL) and washed with a saturated NaHCO_3_ solution (100 mL). The aqueous phase was extracted once more with EtOAc (100 mL) and the combined organic layers were washed with brine (200 mL), dried (Na_2_SO_4_) and evaporated to dryness. The resulting oil was purified by column chromatography, using a mixture of CHCl_3_/MeOH (99/1 to 97/3, *v*/*v*) as the eluent, to afford **8a**, as a mixture with its corresponding *α*-anomer **9a** (280 mg, total yield 53% for two anomers, **8a**:**9a** (3-*β*:*α*) ratio 12:1, as estimated by ^1^H-NMR), **10a** (115 mg, yield 22%) and **11a** (27 mg, yield 5%).

Data for **10a**: Oil, [*α*]_D_ +30.55 (*c*=0.475, CHCl_3_). ^1^H-NMR (600 MHz, CDCl_3_) *δ* 1.32 (t, 3H, CH_3_, *J* = 7.1 Hz), 2.04 (s, 3H, CH_3_CO), 2.17 (s, 3H, CH_3_CO), 2.20 (s, 3H, CH_3_CO), 3.59 (m, 1H, CH_2_), 3.67 (m, 1H, CH_2_), 4.34 (dd, 1H, H-5′, *J*_5′,4′_ = 2.2 Hz, *J*_5′,5′_ = 12.7 Hz), 4.41 (m, 1H, H-4′), 4.65 (dd, 1H, H-5′, *J*_5′,4′_ = 3.8 Hz, *J*_5′,5′_ = 12.7 Hz), 5.31 (dd, 1H, H-3′, *J*_3′,4′_ = 3.6 Hz, *J*_3′,2′_ = 6.2 Hz), 5.35 (m, 1H, H-2′), 5.98 (d, 1H, H-1′, *J*_1′,2′_= 6.5 Hz), 6.13 (br s, 1H, D_2_O exchangeable, NH), 7.52 (s, 1H, H-7). ^13^C-NMR (151 MHz, CDCl_3_) *δ* 15.03 (CH_3_), 20.42 (*CH_3_*CO), 20.69 (*CH_3_*CO), 20.93 (*CH_3_*CO), 38.92 (CH_2_), 62.85 (C-5′), 69.67 (C-3′), 71.61 (C-2′), 81.18 (C-4′), 86.35 (C-1′), 117.71 (C-7), 120.20 (C-6), 125.61 (C-7a), 141.57 (C-5), 153.55 (C-3*α*), 156.43 (C-2), 169.49 (CO), 169.78 (CO), 170.26 (CO). HR-MS (ESI) *m*/*z*: Calcd for C_19_H_22_Cl_2_N_4_O_7_Na: [M + Na]^+^ = 511.0758, found 511.0762.

Data for **11a**: Oil, [*α*]_D_ +61.23 (*c*=0.684, CHCl_3_). ^1^H-NMR (600 MHz, CDCl_3_) *δ* 1.28 (t, 3H, CH_3_, *J* = 7.2 Hz), 1.82 (s, 3H, CH_3_CO), 2.11 (s, 3H, CH_3_CO), 2.15 (s, 3H, CH_3_CO), 3.57 (m, 2H, CH_2_), 4.29 (dd, 1H, H-5′, *J*_5′,4′_ = 4.9 Hz, *J*_5′,5′_ = 12.3 Hz), 4.33 (dd, 1H, H-5′, *J*_5′,4′_ = 3.5 Hz, *J*_5′,5′_ = 12.3 Hz), 4.66 (m, 1H, H-4′), 5.27 (t, 1H, D_2_O exchangeable, NH, *J* = 5.3 Hz), 5.45 (t, 1H, H-3′, *J* = 5.3 Hz), 5.73 (t, 1H, H-2′, *J* = 5.1 Hz), 6.19 (d, 1H, H-1′, *J*_1′,2′_ = 4.9 Hz), 7.55 (s, 1H, H-7). ^13^C-NMR (151 MHz, CDCl_3_) *δ* 14.96 (CH_3_), 20.28 (*CH_3_*CO), 20.55 (*CH_3_*CO), 20.94 (*CH_3_*CO), 38.71 (CH_2_), 63.31 (C-5′), 71.08 (C-3′), 71.55 (C-2′), 78.96 (C-4′), 85.40 (C-1′), 119.37 (C-7), 119.69 (C-6), 125.67 (C-7a), 141.09 (C-5), 154.24 (C-3a), 157.01 (C-2), 169.26 (CO), 169.55 (CO), 170.51 (CO). HR-MS (ESI) *m*/*z*: Calcd for C_19_H_22_Cl_2_N_4_O_7_Na: [M + Na]^+^ = 511.0758, found 511.0764.

5,6-Dichloro-*N*-isopropyl-3-(2′,3′,5′-tri-*O*-acetyl-*β*-d-ribofuranosyl)-3*H*-imidazo[4,5-b]pyridin-2-amine (**8b**), 5,6-dichloro-*N*-isopropyl-3-(2′,3′,5′-tri-*O*-acetyl-*α*-d-ribofuranosyl)-3*H*-imidazo[4,5-b]pyridin-2-amine (**9b**), 5,6-dichloro-*N*-isopropyl-1-(2′,3′,5′-tri-*O*-acetyl-*β*-d-ribofuranosyl)-1*H*-imidazo[4,5-b]pyridin-2-amine (**10b**) and 5,6-dichloro-*N*-isopropyl-1-(2′,3′,5′-tri-*O*-acetyl-*α*-*D*-ribofuranosyl)-1*H*-imidazo[4,5-b]pyridin-2-amine (**11b**): These derivatives were prepared by a procedure analogous to that described for **8a,9a,10a,11a**, starting from imidazopyridine **7b** (280 mg, 1.14 mmol). Purification was effected by column chromatography, using a mixture of CHCl_3_/MeOH (99.5/0.5 to 98/2, *v*/*v*) as the eluent, to afford **8b**, as a mixture with its corresponding *α*-anomer **9b** (350 mg, 61% total yield for two anomers, **8b**:**9b** (3-*β*:*α*) ratio 24:1, as estimated by ^1^H-NMR), **10b** (140 mg, 24% yield) and **11b** (50 mg, 9% yield).

Data for **10b**: Beige solid, mp: 165-166 °C (EtOAc/*n*-pentane). [*α*]_D_ +37.77 (*c*=0.495, CHCl_3_). ^1^H NMR (600 MHz, CDCl_3_) *δ* 1.30 (d, 3H, CH_3_, *J* = 6.5 Hz), 1.31 (d, 3H, CH_3_, *J* = 6.5 Hz), 2.04 (s, 3H, CH_3_CO), 2.15 (s, 3H, CH_3_CO), 2.19 (s, 3H, CH_3_CO), 4.27 (m, 1H, CH), 4.32 (dd, 1H, H-5′, *J*_5′,4′_= 2.2 Hz, *J*_5′,5′_ = 12.7 Hz), 4.39 (m, 1H, H-4′), 4.62 (dd, 1H, H-5′, *J*_5′,4′_ = 4.0 Hz, *J*_5′,5′_ = 12.7 Hz), 5.30 (dd, 1H, H-3′, *J*_3′,4′_ = 3.6 Hz, *J*_3′,2′_ = 6.1 Hz), 5.33 (m, 1H, H-2′), 5.47 (br s, 1H, D_2_O exchangeable, NH), 5.86 (d, 1H, H-1′, *J*_1′,2′_ = 6.7 Hz), 7.53 (s, 1H, H-7). ^13^C-NMR (151 MHz, CDCl_3_) *δ* 20.41 (*CH_3_*CO), 20.67 (*CH_3_*CO), 21.04 (*CH_3_*CO), 22.76 (CH_3_), 23.03 (CH_3_), 46.04 (CH), 62.80 (C-5′), 69.59 (C-3′), 71.87 (C-2′), 81.06 (C-4′), 86.26 (C-1′), 117.74 (C-7), 119.70 (C-6), 125.49 (C-7*α*), 141.38 (C-5), 154.37 (C-3*α*), 156.10 (C-2), 169.44 (CO), 169.72 (CO), 170.29 (CO). HR-MS (ESI) *m*/*z*: Calcd for C_20_H_24_Cl_2_N_4_O_7_Na: [M + Na]^+^ = 525.0914, found 525.0917.

Data for **11b**: Oil, [*α*]_D_ +57.75 (*c* = 0.561, CHCl_3_). ^1^H NMR (400 MHz, CDCl_3_) *δ* 1.25 (d, 3H, CH_3_, *J* = 6.5 Hz), 1.26 (d, 3H, CH_3_, *J* = 6.5 Hz), 1.81 (s, 3H, CH_3_CO), 2.09 (s, 3H, CH_3_CO), 2.14 (s, 3H, CH_3_CO), 4.21 (m, 1H, CH), 4.26 (dd, 1H, H-5′, *J*_5′.4′_ = 5.2 Hz, *J*_5′,5′_ = 12.5 Hz), 4.32 (dd, 1H, H-5′, *J*_5′,4′_ = 3.3 Hz, *J*_5′,5′_ = 12.3 Hz), 4.65 (m, 1H, H-4′), 5.16 (d, 1H, D_2_O exchangeable, NH, *J* = 7.0 Hz), 5.42 (t, 1H, H-3′, *J* = 5.4 Hz), 5.74 (t, 1H, H-2′, *J* = 5.0 Hz), 6.23 (d, 1H, H-1′, *J*_1′,2′_ = 4.7 Hz), 7.55 (s, 1H, H-7). ^13^C-NMR (151 MHz, CDCl_3_) *δ* 20.26 (*CH_3_*CO), 20.53 (*CH_3_*CO), 20.88 (*CH_3_*CO), 22.67 (CH_3_), 23.00 (CH_3_), 45.87 (CH), 63.29 (C-5′), 70.98 (C-3′), 71.48 (C-2′), 78.84 (C-4′), 85.35 (C-1′), 119.32 (C-7), 119.43 (C-6), 125.58 (C-7a), 140.92 (C-5), 154.52 (C-3a), 156.45 (C-2), 169.20 (CO), 169.55 (CO), 170.49 (CO). HR-MS (ESI) *m*/*z*: Calcd for C_20_H_24_Cl_2_N_4_O_7_Na: [M + Na]^+^ = 525.0914, found 525.0917.

*N*-Benzyl-5,6-dichloro-3-(2′,3′,5′-tri-*O*-acetyl-*β*-d-ribofuranosyl)-3*H*-imidazo[4,5-b]pyridin-2-amine (**8c**), *N*-benzyl-5,6-dichloro-3-(2′,3′,5′-tri-*O*-acetyl-*α*-d-ribofuranosyl)-3*H*-imidazo[4,5-b]pyridin-2-amine (**9c**), *N*-benzyl-5,6-dichloro-1-(2′,3′,5′-tri-*O*-acetyl-*β*-d-ribofuranosyl)-1*H*-imidazo[4,5-b]pyridin-2-amine (**10c**) and *N*-benzyl-5,6-dichloro-1-(2′,3′,5′-tri-*O*-acetyl-*α*-d-ribofuranosyl)-1*H*-imidazo[4,5-b]pyridin-2-amine (**11c**): These derivatives were prepared by a procedure analogous to that described for **8a,9a,10a,11a**, starting from imidazopyridine **7c** (350 mg, 1.20mmol). Purification was effected by column chromatography, using a mixture of cyclohexane/EtOAc (70/30 to 20/80, *v*/*v*) as the eluent, to afford **8c**, as a mixture with its corresponding *α*-anomer **9c** (350 mg, 53% total yield for two anomers, **8c**:**9c** (3-*β*:*α*) ratio 12:1, as estimated by ^1^H-NMR), **10c** (100 mg, crude) and **11c** (40 mg, 6% yield). Fractions containing **10c** were pooled and subjected to column chromatography eluted with CHCl_3_/MeOH (99.5/0.5, *v*/*v*), yielding 80 mg of pure **10c** (12% yield).

Data for **10c**: Oil, [*α*]_D_ +46.29 (*c* = 0.337, CHCl_3_). ^1^H NMR (600 MHz, CDCl_3_) *δ* 1.91 (s, 3H, CH_3_CO), 1.99 (s, 3H, CH_3_CO), 2.15 (s, 3H, CH_3_CO), 4.21 (dd, 1H, H-5′, *J*_5′,4′_ = 2.0 Hz, *J*_5′,5′_ = 12.6 Hz), 4.37 (m, 1H, H-4′), 4.57 (dd, 1H, H-5′, *J*_5′,4′_ = 3.4 Hz, *J*_5′,5′_ = 12.6 Hz), 4.72 (dd, 1H, CH_2_, *J*_CH2,NH_ = 5.2 Hz, *J*_CH2,CH2_ = 14.8 Hz), 4.82 (dd, 1H, CH_2_, *J*_CH2,NH_ = 5.9 Hz, *J*_CH2,CH2_ = 14.8 Hz), 5.30 (dd, 1H, H-3′, *J*_3′,4′_ = 3.1 Hz, *J*_3′,2′_ = 6.1 Hz), 5.37 (m, 1H, H-2′), 5.87 (d, 1H, H-1′, *J*_1′,2′_ = 7.6 Hz), 5.88 (br s, 1H, D_2_O exchangeable, NH), 7.27 (m, 1H, phenyl H-4′’), 7.32 (t, 2H, phenyl H-3′’, H-5′’, *J* = 7.5 Hz), 7.37 (d, 2H, phenyl H-2′’, H-6′’, *J* = 7.4 Hz), 7.52 (s, 1H, H-7). ^13^C-NMR (151 MHz, CDCl_3_) *δ* 20.34 (*CH_3_*CO), 20.52 (*CH_3_*CO), 20.70 (*CH_3_*CO), 47.62 (CH_2_), 62.91 (C-5′), 69.83 (C-3′), 71.64 (C-2′), 81.41 (C-4′), 86.04 (C-1′), 117.40 (C-7), 120.01 (C-6), 126.03 (C-7a), 127.89 (phenyl C-2′’, C-6′’), 128.01 (phenyl C-4′’), 128.96 (phenyl C-3′’, C-5′’), 137.97 (phenyl C-1′’), 141.60 (C-5), 154.26 (C-3a), 156.81 (C-2), 169.27 (CO), 169.71 (CO), 170.16 (CO). HR-MS (ESI) *m*/*z*: Calcd for C_24_H_24_Cl_2_N_4_O_7_Na: [M + Na]^+^ = 573.0914, found 573.0919.

Data for **11c**: Oil, [*α*]_D_ +53.17 (*c* = 0.600, CHCl_3_). ^1^H NMR (600 MHz, CDCl_3_) *δ* 1.72 (s, 3H, CH_3_CO), 2.00 (s, 3H, CH_3_CO), 2.12 (s, 3H, CH_3_CO), 4.27 (dd, 1H, H-5′, *J*_5′,4′_ = 4.8 Hz, *J*_5′,5′_ = 12.4 Hz), 4.30 (dd, 1H, H-5′, *J*_5′,4′_ = 3.5 Hz, *J*_5′,5′_ = 12.3 Hz), 4.62 (m, 1H, H-4′), 4.69 (dd, 1H, CH_2_, *J*_CH2,NH_ = 5.0 Hz, *J*_CH2,CH2_ = 14.5 Hz), 4.76 (dd, 1H, CH_2_, *J*_CH2,NH_ = 5.5 Hz, *J*_CH2,CH2_ = 14.5 Hz), 5.44 (t, 1H, H-3′, *J* = 5.2 Hz), 5.60 (br s, 1H, D_2_O exchangeable, NH), 5.71 (t, 1H, H-2′, *J* = 5.2 Hz), 6.19 (d, 1H, H-1′, *J*_1′,2′_ = 5.0 Hz), 7.28 (m, 1H, phenyl H-4′’), 7.32 (m, 2H, phenyl H-3′’, H-5′’), 7.40 (m, 2H, phenyl H-2′’, H-6′’), 7.53 (s, 1H, H-7). ^13^C-NMR (151 MHz, CDCl_3_) *δ* 20.08 (*CH_3_*CO), 20.40 (*CH_3_*CO), 20.94 (*CH_3_*CO), 47.73 (CH_2_), 63.24 (C-5′), 71.03 (C-3′), 71.39 (C-2′), 79.20 (C-4′), 85.46 (C-1′), 118.91 (C-7), 119.87 (C-6), 125.89 (C-7a), 128.02 (phenyl C-4′’), 128.23 (phenyl C-2′’, C-6′’), 128.90 (phenyl C-3′’, C-5′’), 138.03 (phenyl C-1′’), 141.44 (C-5), 154.31 (C-3a), 157.13 (C-2), 169.33 (CO), 169.53 (CO), 170.39 (CO). HR-MS (ESI) *m*/*z*: Calcd for C_24_H_24_Cl_2_N_4_O_7_Na: [M + Na]^+^ = 573.0914, found 573.0920.

5,6-Dichloro-*N*,1-bis-(2′,3′,5′-tri-*O*-acetyl-*β*-d-ribofuranosyl)-1H-imidazo[4,5-b]pyridin-2-amine (**14**), 5,6-dichloro-3-(2′,3′,5′-tri-*O*-acetyl-*β*-d-ribofuranosyl)-3*H*-imidazo[4,5-b]pyridin-2-amine (**8d**) and 5,6-dichloro-1-(2′,3′,5′-tri-*O*-acetyl-*β*-d-ribofuranosyl)-1H-imidazo[4,5-b]pyridin-2-amine (**10d**): These derivatives were prepared by a procedure analogous to that described for **8a** and **10a,** starting from **7d** (245 mg, 1.21 mmol). Purification was effected by column chromatography, using a mixture of CHCl_3_/MeOH (99.5/0.5 to 95.5/4.5, *v*/*v*) as the eluent, to afford **14** (300 mg, 35% yield), **8d** (150 mg, 27% yield) and **10d** (40 mg, 7% yield).

Data for **14**: Oil, [*α*]_D_ −25.60 (*c*=0.703, CHCl_3_). ^1^H NMR (600 MHz, CDCl_3_) *δ* 2.07 (s, 3H, CH_3_CO), 2.07 (s, 3H, CH_3_CO), 2.11 (s, 3H, CH_3_CO), 2.13 (s, 3H, CH_3_CO), 2.14 (s, 3H, CH_3_CO), 2.23 (s, 3H, CH_3_CO), 4.30–4.33 (m, 3H, 2 × H-4′, 1 × H-5′), 4.36 (dd, 1H, H-5′, *J*_5′,4′_ = 5.1 Hz, *J*_5′,5′_ = 12.1 Hz), 4.42 (dd, 1H, H-5′, *J*_5′,4′_ = 3.4 Hz, *J*_5′,5′_ = 12.1 Hz), 4.50 (m, 1H, H-5′), 5.44 (dd, 1H, H-3′, *J*_3′,4′_ = 3.6 Hz, *J*_3′,2′_ = 6.2 Hz), 5.56 (br s, 1H, H-2′), 5.71 (br s, 1H, H-3′), 5.97 (br s, 1H, H-1′), 6.03 (br s, 1H, H-2′), 6.07 (br s, 1H, H-1′), 7.39 (s,1H, H-7). ^13^C-NMR (151 MHz, CDCl_3_) *δ* 20.50 (*CH_3_*CO), 20.60 (*CH_3_*CO), 20.68 (*CH_3_*CO), 20.69 (*CH_3_*CO), 20.90 (*CH_3_*CO), 21.06 (*CH_3_*CO), 63.35 (2xC-5′), 70.04, 70.10, 70.49, 70.77 (2 × C-2′, 2 × C-3′), 79.73 (C-4′), 80.33 (C-4′), 85.06 (C-1′), 85.13 (C-1′), 117.37 (C-7), 122.62, 123.67 (C-6 and C-7a), 139.26, 142.47 (C-3a, C-5), 149.92 (C-2), 169.67 (3 ×CO), 169.76 (CO), 170.37 (CO), 170.65 (CO). HR-MS (ESI) *m*/*z*: Calcd for C_28_H_32_Cl_2_N_4_O_14_Na: [M + Na]^+^ = 741.1184, found 741.1185.

Data for **8d**: Oil, [*α*]_D_ +52.57 (*c*=0.675, CHCl_3_). ^1^H NMR (600 MHz, CDCl_3_) *δ* 2.03 (s, 3H, CH_3_CO), 2.11 (s, 3H, CH_3_CO), 2.16 (s, 3H, CH_3_CO), 4.35 (dd, 1H, H-5′, *J*_5′,4′_ = 2.7 Hz, *J*_5′,5′_ = 12.2 Hz), 4.38 (m, 1H, H-4′), 4.59 (dd, 1H, H-5′, *J*_5′,4′_ = 4.0 Hz, *J*_5′,5′_ = 12.2 Hz), 5.50 (dd, 1H, H-3′, *J*_3′,4′_ = 4.3 Hz, *J*_3′,2′_ = 6.2 Hz), 5.75 (m, 1H, H-2′), 5.87 (br s, 2H, D_2_O exchangeable, NH_2_), 6.23 (d, 1H, H-1′, *J*_1′,2′_ = 6.5 Hz), 7.63 (s, 1H, H-7). ^13^C-NMR (151 MHz, CDCl_3_) *δ* 20.48 (*CH_3_*CO), 20.70 (*CH_3_*CO), 20.83 (*CH_3_*CO), 63.24 (C-5′), 70.27 (C-3′), 71.19 (C-2′), 80.73 (C-4′), 85.04 (C-1′), 123.94 (C-6), 125.17 (C-7), 135.02, 138.38 (C-5 and C-7a), 144.67 (C-3a), 155.30 (C-2), 169.85 (CO), 169.86 (CO), 170.24 (CO). HR-MS (ESI) *m*/*z*: Calcd for C_17_H_18_Cl_2_N_4_O_7_Na: [M + Na]^+^ = 483.0445, found 483.0449.

Data for **10d**: Oil, [*α*]_D_ +22.70 (*c* = 0.185, CHCl_3_). ^1^H NMR (600 MHz, CDCl_3_) *δ* 2.02 (s, 3H, CH_3_CO), 2.16 (s, 3H, CH_3_CO), 2.35 (s, 3H, CH_3_CO), 4.31 (dd, 1H, H-5′, *J*_5′,4′_ = 2.1 Hz, *J*_5′,5′_ = 12.5 Hz), 4.43 (m, 1H, H-4′), 4.65 (dd, 1H, H-5′, *J*_5′,4′_ = 1.9 Hz, *J*_5′,5′_ = 12.5 Hz), 5.39 (dd, 1H, H-3′, *J*_3′,4′_ = 3.2 Hz, *J*_3′,2′_ = 6.2 Hz), 5.47 (m, 1H, H-2′), 5.89 (d, 1H, H-1′, *J*_1′,2′_ = 7.5 Hz), 7.47 (br s, 2H, D_2_O exchangeable, NH_2_), 7.52 (s, 1H, H-7). ^13^C-NMR (151 MHz, CDCl_3_) *δ* 20.36 (*CH_3_*CO), 20.65 (*CH_3_*CO), 21.23 (*CH_3_*CO), 63.01 (C-5′), 70.07 (C-3′), 71.20 (C-2′), 81.20 (C-4′), 86.11 (C-1′), 117.54 (C-7), 119.80 (C-6), 125.35 (C-7a), 141.36 (C-5), 153.77 (C-3a), 157.58 (C-2), 169.30 (CO), 169.71 (CO), 170.43 (CO). HR-MS (ESI) *m*/*z*: Calcd for C_17_H_18_Cl_2_N_4_O_7_Na: [M + Na]^+^ = 483.0445, found 483.0440.

5,6-Dichloro-*N*-ethyl-3-(*β*-d-ribofuranosyl)-3*H*-imidazo[4,5-b]pyridin-2-amine (**12a**): A mixture of **8a** and **9a** (80 mg, 0.16 mmol) was treated for 18 h with a saturated methanolic ammonia solution (15 mL). Upon evaporation of the solvent, the residue was purified by column chromatography using a mixture of EtOAc/MeOH: 99/1 (*v*/*v*), as the eluent and the desired product was recrystallized from methanol to provide pure **12a** (35 mg, 59% yield). White solid, mp: 191–192 °C (MeOH). [*α*]_D_ +31.88 (*c* = 0.367, MeOH). ^1^H NMR (600 MHz, DMSO-*d*_6_) *δ* 1.19 (t, 3H, CH_3_, *J* = 7.2 Hz), 3.41 (m, 2H, CH_2_, overlapping with water of DMSO-*d*_6_), 3.66 (m, 2H, H-5′), 3.99 (m, 1H, H-4′), 4.12 (dd, 1H, H-3′, *J*_3′,4′_ = 1.5 Hz, *J*_3′,2′_ = 5.2 Hz), 4.59 (dd, 1H, H-2′, *J*_2′,3′_ = 5.2 Hz, *J*_2′,1′_ = 7.3 Hz), 5.05–5.55 (br s, 2H, D_2_O exchangeable, 2 × OH), 5.75 (br s, 1H, D_2_O exchangeable, OH), 5.96 (d, 1H, H-1′, *J*_1′,2′_ = 7.5 Hz), 7.64 (br s, 1H, D_2_O exchangeable, NH), 7.78 (s, 1H, H-7). ^13^C-NMR (151 MHz, DMSO-*d*_6_) *δ* 14.50 (CH_3_), 37.08 (CH_2_), 61.42 (C-5′), 70.29 (C-2′), 71.04 (C-3′), 85.74 (C-4′), 86.37 (C-1′), 121.03 (C-6), 122.82 (C-7), 134.65, 136.07 (C-5 and C-7a), 145.96 (C-3a), 155.48 (C-2). HR-MS (ESI) *m*/*z*: Calcd for C_13_H_17_Cl_2_N_4_O_4_: [M + H]^+^ = 363.0621, found 363.0615.

5,6-Dichloro-*N*-isopropyl-3-(*β*-d-ribofuranosyl)-3*H*-imidazo[4,5-b]pyridin-2-amine (**12b**): This compound was prepared by a procedure analogous to that described for **12a** starting from **8b** (90 mg, 0.18mmol, containing also the corresponding *α*-anomer **9b**). Purification was effected by column chromatography using a mixture of DCM/MeOH: 98/2 to 95/5 (*v*/*v*) as the eluent, to afford **12b** (55 mg, 81% yield). White solid, mp: 127–128 °C (EtOAc/*n*-pentane). [*α*]_D_ +30.77 (*c* = 0.494, MeOH). ^1^H NMR (600 MHz, DMSO-*d*_6_) *δ* 1.21 (d, 3H, CH_3_, *J* = 6.5 Hz), 1.22 (d, 3H, CH_3_, *J* = 6.5 Hz), 3.65 (dd, 1H, H-5′, *J*_5′,4′_ = 2.8 Hz, *J*_5′,5′_ = 11.7 Hz), 3.68 (dd, 1H, H-5′, *J*_5′,4′_ = 2.0 Hz, *J*_5′,5′_ = 11.7 Hz), 3.99 (m, 1H, H-4′), 4.11–4.18 (m, 2H, H-3′, CH), 4.56 (dd, 1H, H-2′, *J*_2′,3′_ = 5.4 Hz, *J*_2′,1′_ = 7.6 Hz), 5.02–5.52 (br s, 2H, D_2_O exchangeable, 2 × OH), 5.72 (br s, 1H, D_2_O exchangeable, OH), 5.97 (d, 1H, H-1′, *J*_1′,2′_ = 7.6 Hz), 7.44 (d, 1H, D_2_O exchangeable, NH, *J* = 7.5 Hz), 7.78 (s, 1H, H-7). ^13^C-NMR (151 MHz, DMSO-*d*_6_) *δ* 22.23 (CH_3_), 22.37 (CH_3_), 44.36 (CH), 61.42 (C-5′), 70.25 (C-2′), 71.03 (C-3′), 85.71 (C-4′), 86.26 (C-1′), 121.00 (C-6), 122.72 (C-7), 134.60, 135.99 (C-5 and C-7a), 145.91 (C-3a), 154.84 (C-2). HR-MS (ESI) *m*/*z*: Calcd for C_14_H_19_Cl_2_N_4_O_4_: [M + H]^+^ = 377.0778, found 377.0786.

*N*-Benzyl-5,6-dichloro-3-(*β*-d-ribofuranosyl)-3*H*-imidazo[4,5-b]pyridin-2-amine (**12c**): This compound was prepared by a procedure analogous to that described for **12a** starting from **8c** (130 mg, 0.23 mmol, containing also the corresponding *α*-anomer **9c**). Purification was effected by column chromatography using a mixture of DCM/MeOH: 98/2 to 90/10 (*v*/*v*) to afford an anomeric mixture *β*/*α*, in a 12/1 ratio (80 mg). A second column chromatography was performed (DCM/MeOH: 97.5/2.5 to 90/10, *v*/*v*) and the fractions pooled (30 mg) were enriched in the desired *β*-anomer **12c** (*β*/*α*ratio: 17/1). From this mixture, pure **12c** (12 mg) was obtained by semi-preparative HPLC, eluted with H_2_O (+0.2% Acetic Acid)/ACN (70/30 to 60/40, *v*/*v*, over a period of 40 min); t_R_=23.96 min. Oil, [*α*]_D_ +6.44 (*c*=0.652, MeOH). ^1^H NMR (600 MHz, DMSO-*d6*) *δ* 3.67 (br s, 2H, H-5′), 4.02 (m, 1H, H-4′), 4.14 (m, 1H, H-3′), 4.58 (dd, 1H, CH_2_, *J*_CH2,NH_ = 5.8 Hz, *J*_CH2,CH2_ = 15.6 Hz), 4.63 (dd, 1H, CH_2_, *J*_CH2,NH_ = 6.4 Hz, *J*_CH2,CH2_ = 15.6 Hz), 4.67 (m, 1H, H-2′), 5.22 (d, 1H, D_2_O exchangeable, OH-3′, *J* = 4.1 Hz), 5.40 (d, 1H, D_2_O exchangeable, OH-2′, *J* = 6.3 Hz), 5.72 (t, 1H, D_2_O exchangeable, OH-5′, *J* = 4.3 Hz), 6.01 (d, 1H, H-1′, *J*_1′,2′_ = 7.5 Hz), 7.23 (t, 1H, phenyl H-4′’, *J* = 7.1 Hz), 7.32 (t, 2H, phenyl H-3′’, H-5′’, *J* = 7.6 Hz), 7.35 (d, 2H, phenyl H-2′’, H-6′’, *J* = 7.2 Hz), 7.78 (s, 1H, H-7), 8.25 (t, 1H, D_2_O exchangeable, NH, *J* = 6.1 Hz). ^13^C-NMR (151 MHz, DMSO-*d*_6_) *δ* 45.14 (CH_2_), 61.45 (C-5′), 70.53 (C-2′), 71.05 (C-3′), 85.80 (C-4′), 86.46 (C-1′), 121.08 (C-6), 123.09 (C-7), 126.79 (phenyl C-4′’), 126.99 (phenyl C-2′’, C-6′’), 128.26 (phenyl C-3′’, C-5′’), 134.73, 136.10 (C-5 and C-7a), 139.25 (phenyl C-1′’), 146.01 (C-3a), 155.69 (C-2). HR-MS (ESI) *m*/*z*: Calcd for C_18_H_17_Cl_2_N_4_O_4_: [M − H]^−^ = 423.0632, found 423.0632.

5,6-Dichloro-3-(*β*-d-ribofuranosyl)-3*H*-imidazo[4,5-b]pyridin-2-amine (**12d**)*:* This compound was prepared by a procedure analogous to that described for **12a**, starting from **8d** (100 mg, 0.22 mmol). Purification was effected by column chromatography using a mixture of DCM/MeOH: 97/3 to 90/10 (*v*/*v*) as the eluent, to result in **12d** (70 mg, 96% yield). Beige solid, mp: 142–143 °C (toluene). [*α*]_D_ +16.50 (*c* = 0.594, MeOH). ^1^HNMR (600 MHz, DMSO-*d*_6_) *δ* 3.64 (brs, 2H, H-5′), 3.98 (m, 1H, H-4′), 4.12 (m, 1H, H-3′), 4.62 (m, 1H, H-2′), 5.20 (br s, 1H, D_2_O exchangeable, OH-3′), 5.35 (brs, 1H, D_2_O exchangeable, OH-2′), 5.58 (brs, 1H, D_2_O exchangeable, OH-5′), 5.94 (d, 1H, H-1′, *J*_1′,2′_ = 7.4 Hz), 7.39 (brs, 2H, D_2_O exchangeable, NH_2_), 7.71 (s, 1H, H-7). ^13^C-NMR (151 MHz, DMSO-*d*_6_) *δ* 61.44 (C-5′), 70.31 (C-2′), 70.95 (C-3′), 85.73 (C-4′), 86.37 (C-1′), 121.12 (C-6), 122.58 (C-7), 134.39, 136.37 (C-5 and C-7a), 145.71 (C-3a), 156.45 (C-2). HR-MS (ESI) *m*/*z*: Calcd for C_11_H_13_Cl_2_N_4_O_4_: [M + H]^+^ = 335.0308, found 335.0317.

5,6-Dichloro-*N*-ethyl-1-(*β*-d-ribofuranosyl)-1*H*-imidazo[4,5-b]pyridin-2-amine (**13a**)*:* This compound was prepared by a procedure analogous to that described for **12a** starting from **10a** (50 mg, 0.10 mmol). Purification was effected by column chromatography using a mixture of DCM/MeOH: 98/2 to 85/15 (*v*/*v*) as the eluent to afford **13a** (30 mg, 81% yield). White solid, mp: 226-227 °C (MeOH). [*α*]_D_ +47.23 (*c* = 0.271, MeOH). ^1^HNMR (600 MHz, DMSO-*d*_6_) *δ* 1.18 (t, 3H, CH_3_, *J* = 7.2 Hz), 3.41 (m, 2H, CH_2_, overlapping with water of DMSO-*d*_6_), 3.66 (dd, 1H, H-5′, *J*_5′,4′_ = 2.0 Hz, *J*_5′,5′_ = 11.8 Hz), 3.71 (m, 1H, H-5′), 4.00 (m, 1H, H-4′), 4.08 (dd, 1H, H-3′, *J*_3′,4′_ = 1.6 Hz, *J*_3′,2_′ = 5.5 Hz), 4.28 (m, 1H, H-2′), 5.22–5.37 (brs, 2H, D_2_O exchangeable, 2 × OH), 5.73 (brs, 1H, D_2_O exchangeable, OH), 5.77 (d, 1H, H-1′, *J*_1′,2′_ = 7.7 Hz), 7.61 (t, 1H, D_2_O exchangeable, NH, *J* = 5.3 Hz), 8.04 (s, 1H, H-7). ^13^C-NMR (151 MHz, DMSO-*d*_6_) *δ* 14.55 (CH_3_), 37.52 (CH_2_), 61.09 (C-5′), 70.26 (C-3′), 71.49 (C-2′), 86.00 (C-4′), 87.55 (C-1′), 117.00 (C-6), 118.13 (C-7), 126.68 (C-7a), 138.26 (C-5), 154.74 (C-3a), 157.22 (C-2). HR-MS (ESI) *m*/*z*: Calcd for C_13_H_17_Cl_2_N_4_O_4_: [M + H]^+^ = 363.0621, found 363.0630.

5,6-Dichloro-*N*-isopropyl-1-(*β*-d-ribofuranosyl)-1*H*-imidazo[4,5-b]pyridin-2-amine (**13b**):This compound was prepared by a procedure analogous to that described for **12a** starting from **10b** (90 mg, 0.18 mmol). Purification was effected by column chromatography, using a mixture of DCM/MeOH: 97/3 to 88/12 (*v*/*v*) as the eluent, to afford **13b** (65 mg, 96% yield). White solid, mp: 191–192 °C (EtOAc/*n*-pentane). [*α*]_D_ +45.80 (*c* = 0.559, MeOH). ^1^H NMR (600 MHz, DMSO-*d*_6_) *δ* 1.22 (d, 6H, 2 × CH_3_, *J* = 6.6 Hz), 3.65 (ddd, 1H, H-5′, *J*_5′,4′_ = 2.5 Hz, *J*_5′,OH_ = 4.4 Hz, *J*_5′,5′_ = 11.9 Hz), 3.71 (m, 1H, H-5′), 4.00 (m, 1H, H-4′), 4.08 (m, 1H, H-3′), 4.11 (m, 1H, CH), 4.27 (m, 1H, H-2′), 5.27 (d, 1H, D_2_O exchangeable, OH-3′, *J* = 3.9 Hz), 5.30 (d, 1H, D_2_O exchangeable, OH-2′, *J* = 7.6 Hz), 5.70 (t, 1H, D_2_O exchangeable, OH-5′, *J* = 4.4 Hz), 5.78 (d, 1H, H-1′, *J*_1′,2′_ = 7.8 Hz), 7.37 (d, 1H, D_2_O exchangeable, NH, *J* = 7.7 Hz), 8.02 (s, 1H, H-7). ^13^C-NMR (151 MHz, DMSO-*d*_6_) *δ* 22.23 (CH_3_), 22.33 (CH_3_), 44.87 (CH), 61.11 (C-5′), 70.26 (C-3′), 71.49 (C-2′), 86.02 (C-4′), 87.53 (C-1′), 116.90 (C-6), 117.96 (C-7), 126.73 (C-7a), 138.22 (C-5), 154.76 (C-3a), 156.63 (C-2). HR-MS (ESI) *m*/*z*: Calcd for C_14_H_19_Cl_2_N_4_O_4_: [M + H]^+^ = 377.0778, found 377.0785.

*N*-Benzyl-5,6-dichloro-1-(*β*-d-ribofuranosyl)-1*H*-imidazo[4,5-b]pyridin-2-amine (**13c**): This compound was prepared by a procedure analogous to that described for **12a** starting from **10c** (60 mg, 0.11 mmol). Purification was effected by column chromatography, using a mixture of DCM/MeOH: 97.5/2.5 to 85/15 (*v*/*v*) as the eluent to afford **13c** (40 mg, 90% yield).White solid, mp: 136-137 °C (toluene). [*α*]_D_ +17.37 (*c* = 0.570, MeOH). ^1^H NMR (600 MHz, DMSO-*d*_6_) *δ*3.66 (dd, 1H, H-5′, *J*_5′,4′_ = 2.5 Hz, *J*_5′,5′_ = 11.9 Hz), 3.72 (dd, 1H, H-5′, *J*_5′,4′_ = 1.8 Hz, *J*_5′,5′_ = 11.9 Hz), 4.03 (m, 1H, H-4′), 4.09 (dd, 1H, H-3′, *J*_3′,4′_ = 1.6 Hz, *J*_3′,2′_ = 5.4 Hz), 4.32 (dd, 1H, H-2′, *J*_2′,3′_ = 5.5 Hz, *J*_2′,1′_ = 7.8 Hz), 4.59 (d, 2H, CH_2_, *J*_CH2,NH_ = 6.1 Hz), 5.07–5.57 (br s, 2H, D_2_O exchangeable, 2 × OH), 5.77 (br s, 1H, D_2_O exchangeable, OH), 5.83 (d, 1H, H-1′, *J*_1′,2′_ = 7.8 Hz), 7.24 (t, 1H, phenyl H-4′’, *J* = 7.2 Hz), 7.32 (t, 2H, phenyl H-3′’, H-5′’, *J* = 7.6 Hz), 7.36 (d, 2H, phenyl H-2′’, H-6′’, *J* = 7.1 Hz), 8.09 (s, 1H, H-7), 8.26 (t, 1H, D_2_O exchangeable, NH, *J*_NH,CH2_ = 6.1 Hz). ^13^C-NMR (151 MHz, DMSO-*d*_6_) *δ* 45.71 (CH_2_), 61.11 (C-5′), 70.26 (C-3′), 71.67 (C-2′), 86.10 (C-4′), 87.55 (C-1′), 117.22 (C-6), 118.50 (C-7), 126.64 (C-7a), 126.87 (phenyl C-4′), 127.21 (phenyl C-2′’, C-6′’), 128.26 (phenyl C-3′’, C-5′’), 138.40 (C-5), 139.24 (phenyl C-1′’), 154.43 (C-3a), 157.31 (C-2). HR-MS (ESI) *m*/*z*: Calcd for C_18_H_19_Cl_2_N_4_O_4_: [M + H]^+^ = 425.0778, found 425.0784.

5,6-Dichloro-1-(*β*-d-ribofuranosyl)-1*H*-imidazo[4,5-b]pyridin-2-amine (**13d**): This compound was prepared by a procedure analogous to that described for **12a** starting from **10d** (40 mg, 0.09 mmol). Purification was effected by column chromatography, using a mixture of DCM/MeOH: 97/3 to 88/12 (*v*/*v*) as the eluent, to give **13d** (25 mg, 86% yield). Beige solid, mp: 194–195 °C (toluene). [*α*]_D_ +19.44 (*c* = 0.360, MeOH). ^1^H NMR (600 MHz, DMSO-*d*_6_) *δ* 3.64 (m, 1H, H-5′), 3.70 (m, 1H, H-5′), 3.99 (m, 1H, H-4′), 4.08 (dd, 1H, H-3′, *J*_3′,4′_ = 1.5 Hz, *J*_3′,2′_ = 5.5 Hz), 4.29 (m, 1H, H-2′), 5.25 (br s, 1H, D_2_O exchangeable, OH-3′), 5.30 (br s, 1H, D_2_O exchangeable, OH-2′), 5.60 (br s, 1H, D_2_O exchangeable, OH-5′), 5.76 (d, 1H, H-1′, *J*_1′,2′_ = 7.7 Hz), 7.45 (br s, 2H, D_2_O exchangeable, NH_2_), 8.08 (s, 1H, H-7). ^13^C-NMR (151 MHz, DMSO-*d6*) *δ* 61.01 (C-5′), 70.07 (C-3′), 71.56 (C-2′), 85.96 (C-4′), 87.60 (C-1′), 116.70 (C-6), 118.29 (C-7), 126.13 (C-7a), 138.41 (C-5), 154.85 (C-3a), 158.06 (C-2). HR-MS (ESI) *m*/*z*: Calcd for C_11_H_13_Cl_2_N_4_O_4_: [M + H]^+^ = 335.0308, found 335.0318.

### 3.3. Antiviral Evaluation

The compounds were evaluated against different herpesviruses, including herpes simplex virus type 1 (HSV-1) strain KOS, a thymidine kinase-deficient (TK^–^) HSV-1 KOS strain that is resistant to ACV (ACV^r^), herpes simplex virus type 2 (HSV-2) strain G, adeno virus-2, human coronavirus, varicella-zoster virus (VZV) TK^+^ strain Oka, TK^–^ VZV strain 07-1, and human cytomegalovirus (HCMV) strains AD-169 and Davis. The antiviral assays were based on inhibition of virus-induced cytopathicity or plaque formation (for VZV) in human embryonic lung (HEL) fibroblasts. Confluent cell cultures in microtiter 96-well plates were inoculated with 100 CCID_50_ of virus (1 CCID_50_ being the virus dose to infect 50% of the cell cultures) or with 20 plaque forming units (PFU) (for VZV) and the cell cultures were incubated in the presence of varying concentrations of the test compounds. Viral cytopathicity or plaque formation (VZV) was recorded as soon as it reached completion in the control virus-infected cell cultures that were not treated with the test compounds. Antiviral activity was expressed as the EC_50_ or compound concentration required, to reduce virus-induced cytopathicity or viral plaque formation by 50%. Cytotoxicity of the test compounds was expressed as the minimum cytotoxic concentration (MCC) or the compound concentration that caused a microscopically detectable alteration of cell morphology. Alternatively, the cytostatic activity of the test compounds was measured based on inhibition of cell growth. HEL cells were seeded at a rate of 5 × 10^3^ cells/well into 96-well microtiter plates and allowed to proliferate for 24 h. Then, medium containing different concentrations of the test compounds was added. After 3 days of incubation at 37 °C, the cell number was determined with a Coulter counter. The cytostatic concentration was calculated as the CC50, or the compound concentration required to reduce cell proliferation by 50% relative to the number of cells in the untreated controls.

### 3.4. Proliferation Assay

The human cell lines used for the proliferation were Hela (ATCC #CCL-2, cervical carcinoma), CEM (T-lymphoblastoid cells) and HMEC-1 (human microvascular endothelialcells). First, (5−7.5) × 10^4^ cells were seeded onto standard 96-well microtiter plates and left to attach for 24 h. On the next day, test compounds were added in five serial 10-fold dilutions. The cell growth rate was evaluated after 72 h of incubation, using MTT assay. Obtained results are expressed as an IC_50_ value, which stands for the concentration of the compound necessary for 50% growth inhibition. The IC_50_ values are calculated from concentration–response curve using linear regression analysis. Each test was performed in quadruplicate in at least two individual experiments.

## Supplementary Materials

The following are available online: a figure indicating the numbering of the imidazopyridine nucleosides (Figure S1); ^1^H- and ^13^C-NMR spectra of the target nucleosides **12a**–**d** and **13a**–**d**, and of the di-ribosylated by-product **14** (Figures S2–S10); the NOE spectra of target compounds **12a** and **13a** (Figures S11 and S12); tables with the results of the antiviral (Tables S1–S3) and the cytotoxic (Table S4) evaluation of the target nucleosides.
