# Supplementary material for: Synthesis of New Imidazopyridine Nucleoside Derivatives Designed as Maribavir Analogues
Source: Molecules. 2020 Oct 3;25(19):4531. doi: 10.3390/molecules25194531 (PMC7582934; doi:10.3390/molecules25194531)
Supplement: Supplementary file 1 [file molecules-25-04531-s001.pdf]

Supplementary Material

SYNTHESIS OF NEW IMIDAZOPYRIDINE NUCLEOSIDE DERIVATIVES DESIGNED AS MARIBAVIR ANALOGUES

Georgios Papadakis [1], Maria Gerasi [1], Robert Snoeck [2], Panagiotis Marakos [1], Graciela Andrei [2], Nikolaos Lougiakis [1*] and Nicole Pouli [1].

[1] Division of Pharmaceutical Chemistry, Department of Pharmacy, National and Kapodistrian University of Athens, Panepistimiopolis-Zografou, Athens 15771, Greece; gpapadakis@pharm.uoa.gr (G.P.); mairager@pharm.uoa.gr (M.G.); marakos@pharm.uoa.gr (P.M.); pouli@pharm.uoa.gr (N.P.)

[2] Laboratory of Virology & Chemotherapy, Rega Institute, KU Leuven, B-3000 Leuven, Belgium; robert.snoeck@kuleuven.be (R.S.); graciela.andrei@kuleuven.be (G.A.)

[*] Correspondence: nlougiak@pharm.uoa.gr

Table of contents

1. Figure S1: Numbering of the imidazo[4,5-*b*]pyridine nucleosides. S-2
2. Figure S2: ^{1}H- and ^{13}C-NMR spectra of compound **12a**. S-3
3. Figure S3: ^{1}H- and ^{13}C-NMR spectra of compound **12b**. S-4
4. Figure S4: ^{1}H- and ^{13}C-NMR spectra of compound **12c**. S-5
5. Figure S5: ^{1}H- and ^{13}C-NMR spectra of compound **12d**. S-6
6. Figure S6: ^{1}H- and ^{13}C-NMR spectra of compound **13a**. S-7
7. Figure S7: ^{1}H- and ^{13}C-NMR spectra of compound **13b**. S-8
8. Figure S8: ^{1}H- and ^{13}C-NMR spectra of compound **13c**. S-9
9. Figure S9: ^{1}H- and ^{13}C-NMR spectra of compound **13d**. S-10
10. Figure S10: ^{1}H- and ^{13}C-NMR spectra of compound **14**. S-11
11. Figure S11: NOE spectrum of compound **12a**. S-12
12. Figure S12: NOE spectrum of compound **13a**. S-13
13. Table S1: Antiviral activity of the target compounds against HSV-1, HSV-2, Vaccinia virus, Adeno virus-2 and Human Coronavirus (229E). S-14
14. Table S2: Antiviral activity of the target compounds against varicella-zoster virus (VZV). S-14
15. Table S3: Antiviral activity of the target compounds against human cytomegalovirus (HCMV). S-15
16. Table S4: Inhibitory effects of the target compounds on the proliferation of human T-lymphocyte cells (CEM), human cervix carcinoma cells (HeLa) and human dermal microvascular endothelial cells (HMEC-1). S-15

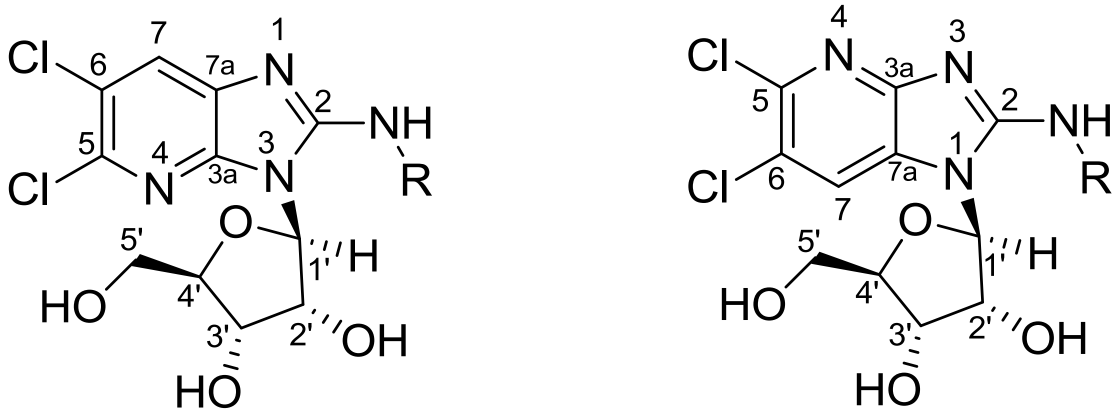

Figure S1: Numbering of the imidazo[4,5-*b*]pyridine nucleosides.

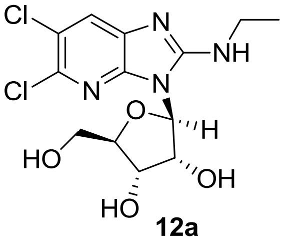

12a

^{1}H-NMR

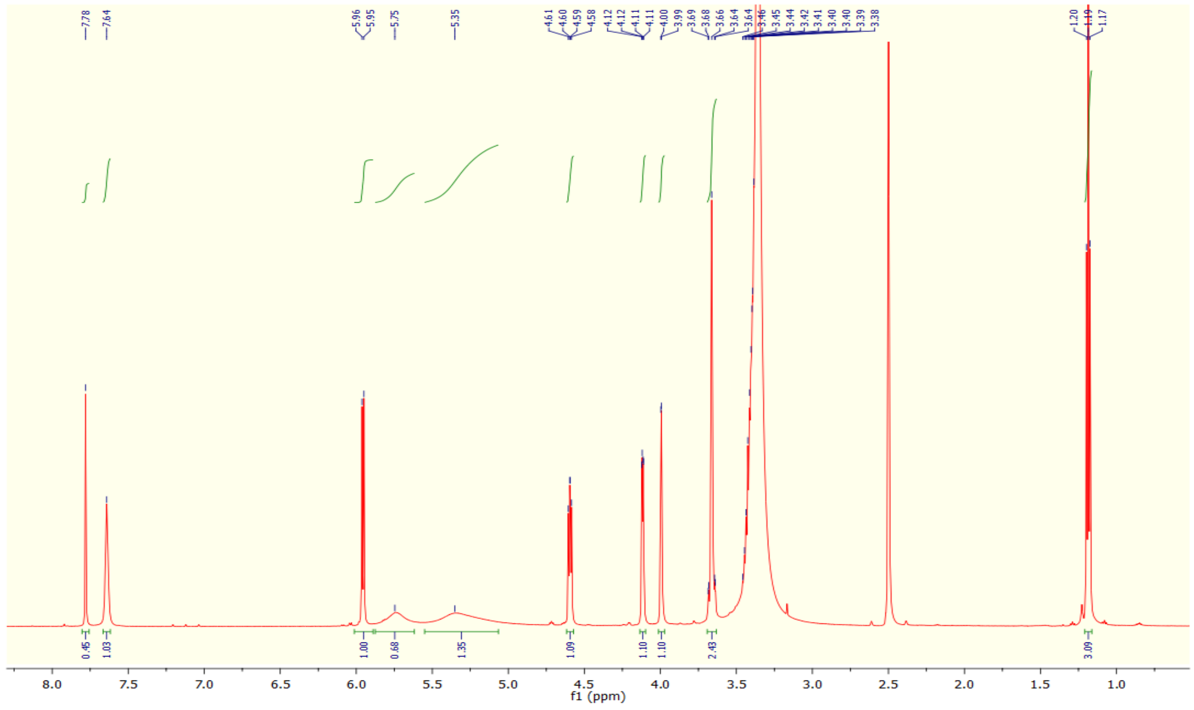

^{13}C-NMR

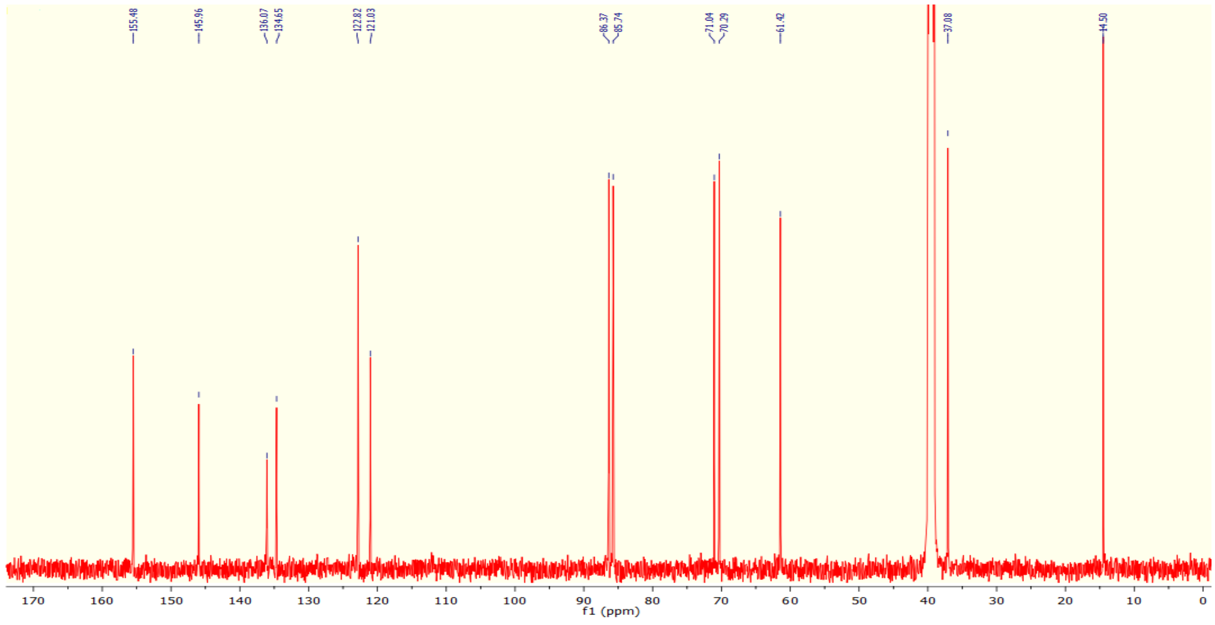

Figure S2: ^{1}H- and ^{13}C-NMR spectra of compound **12a**.

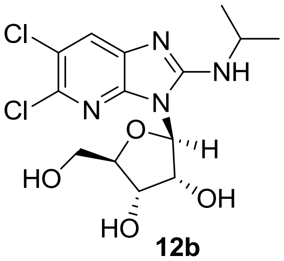

12b

^{1}H-NMR

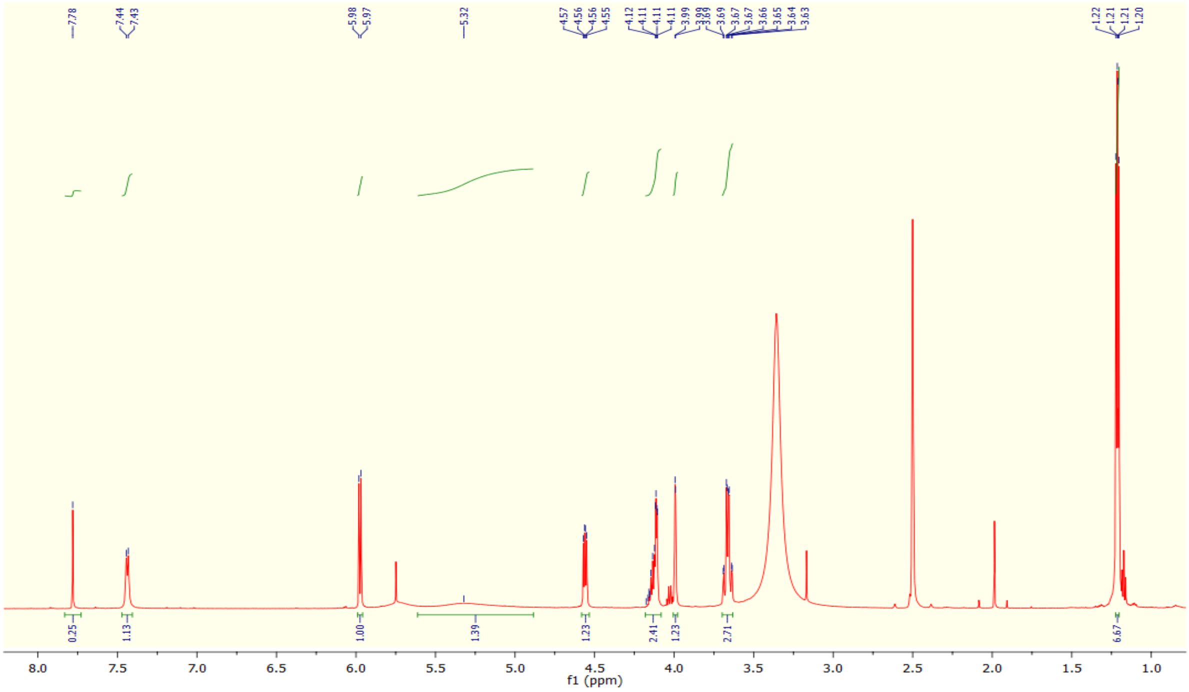

^{13}C-NMR

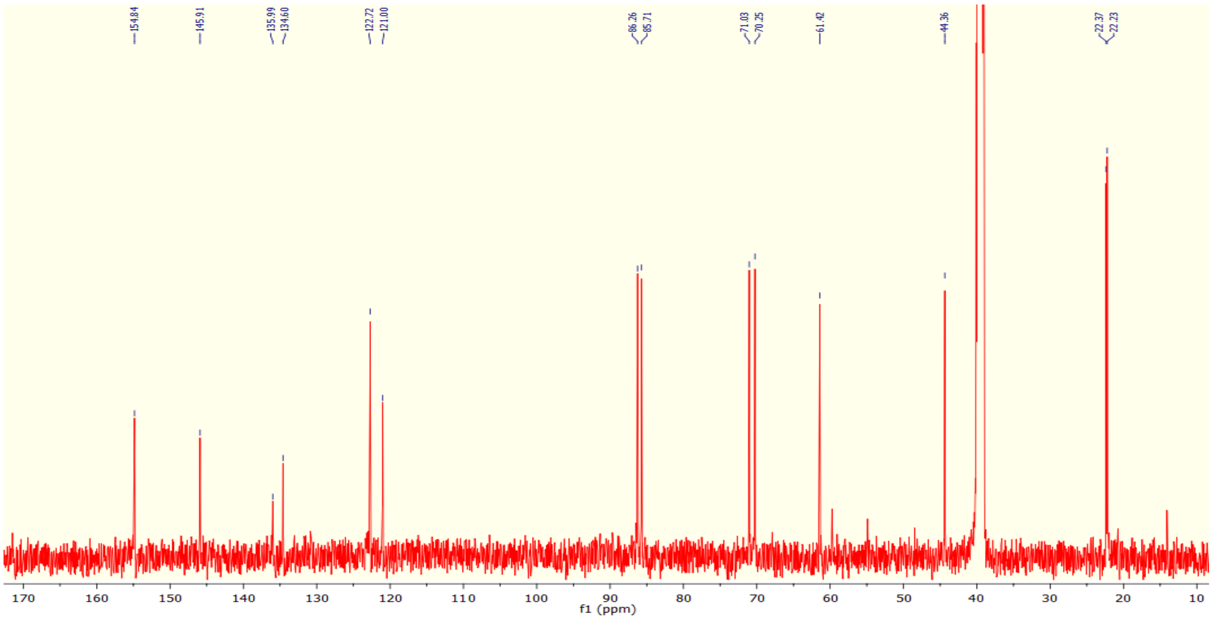

Figure S3: ^{1}H- and ^{13}C-NMR spectra of compound **12b**.

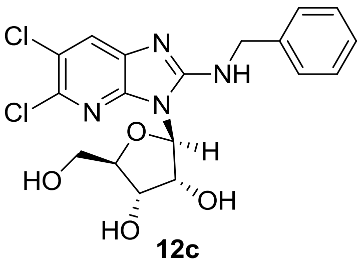

12c

^{1}H-NMR

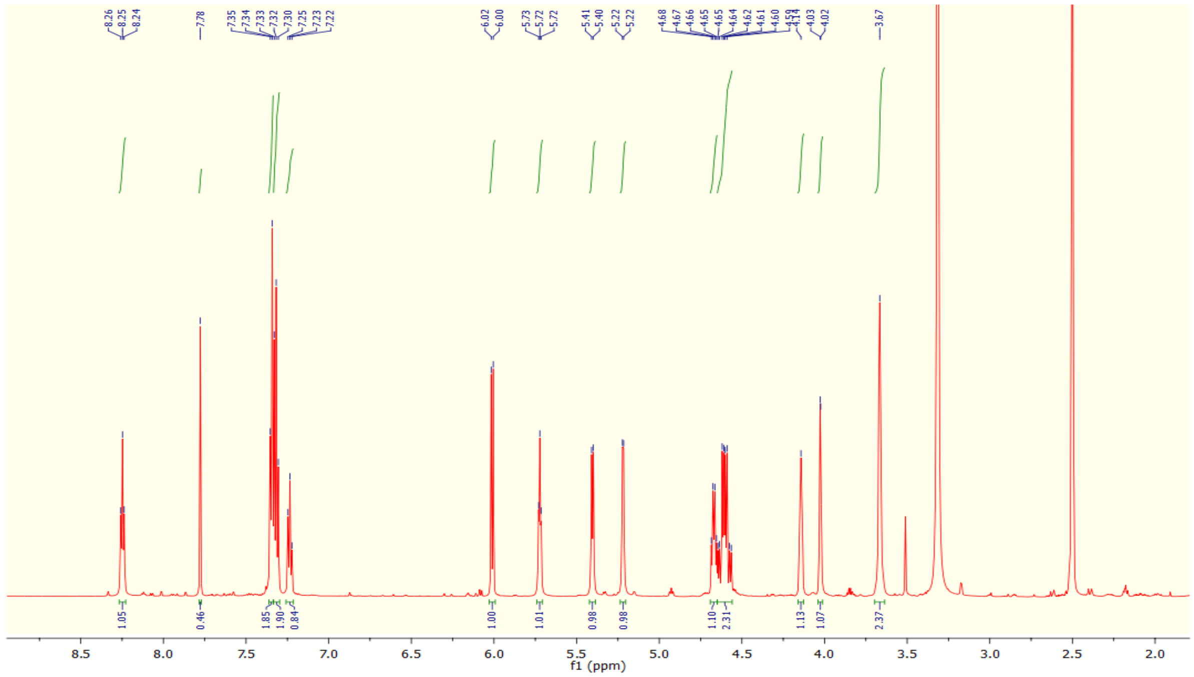

^{13}C-NMR

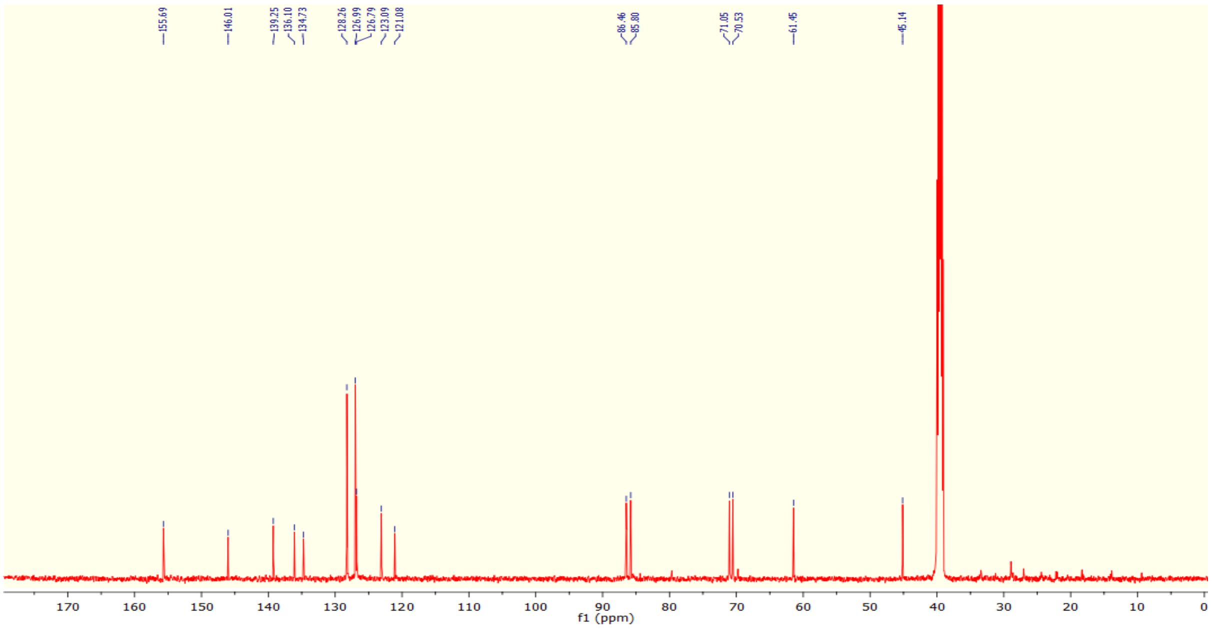

Figure S4: ^{1}H- and ^{13}C-NMR spectra of compound **12c**.

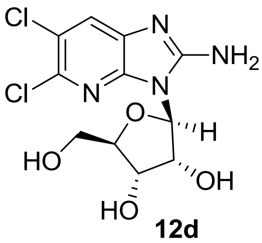

12d

^{1}H-NMR

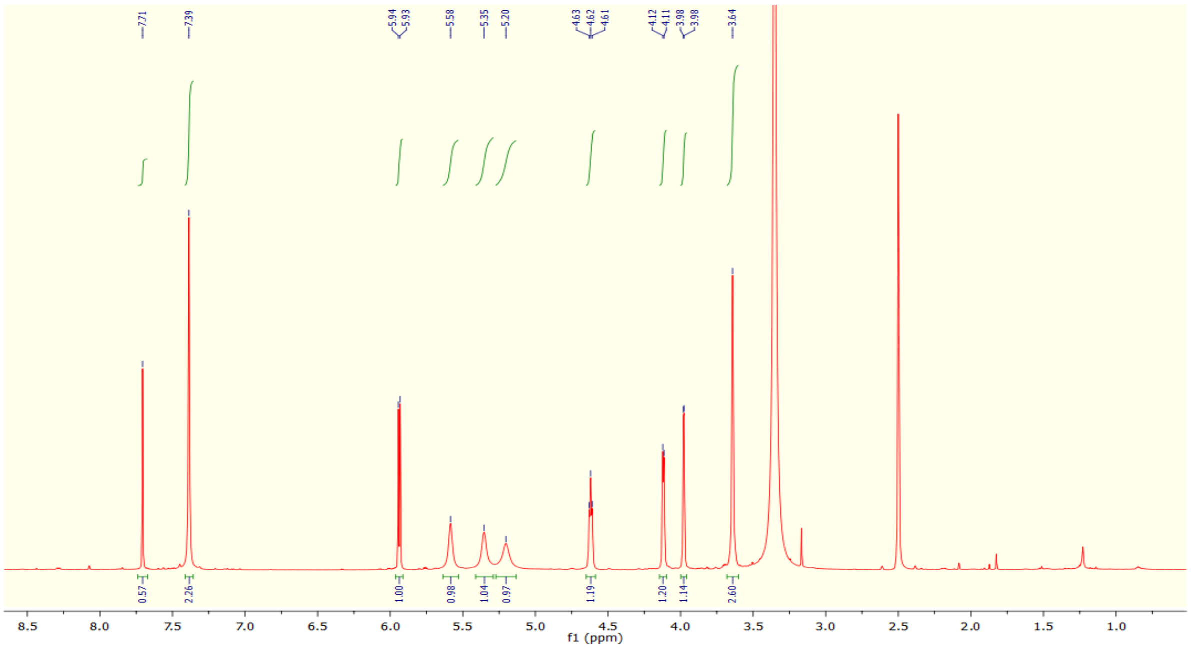

^{13}C-NMR

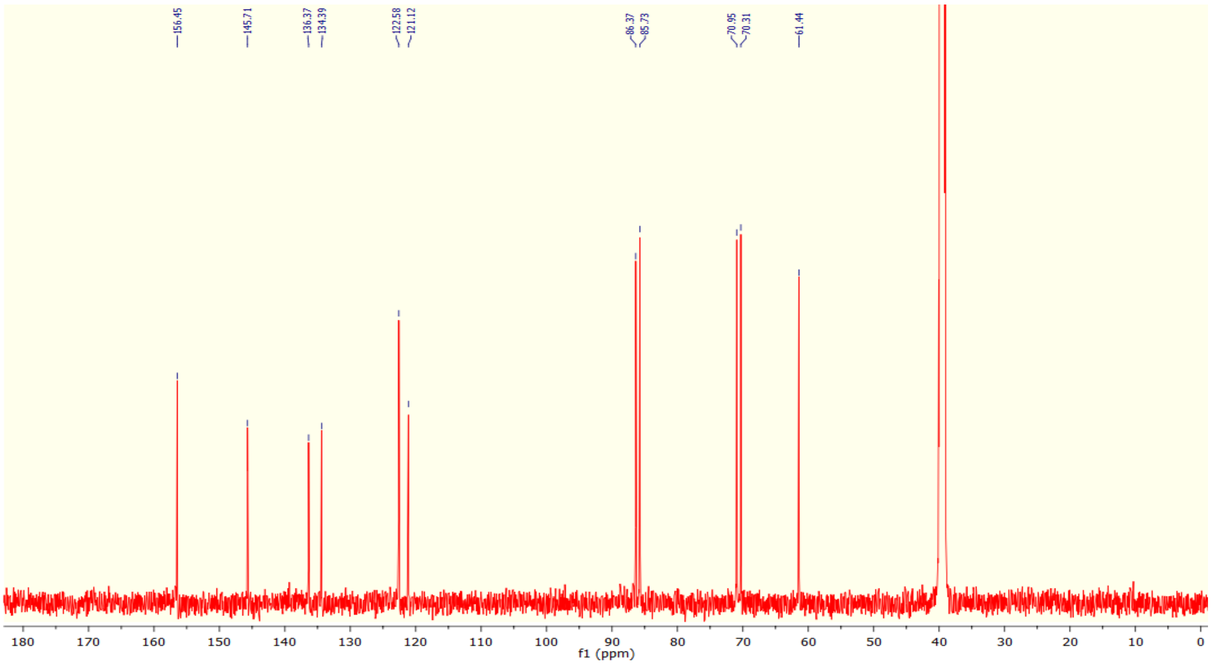

Figure S5: ^{1}H- and ^{13}C-NMR spectra of compound **12d**.

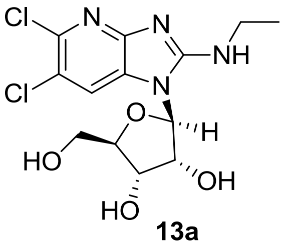

13a

^{1}H-NMR

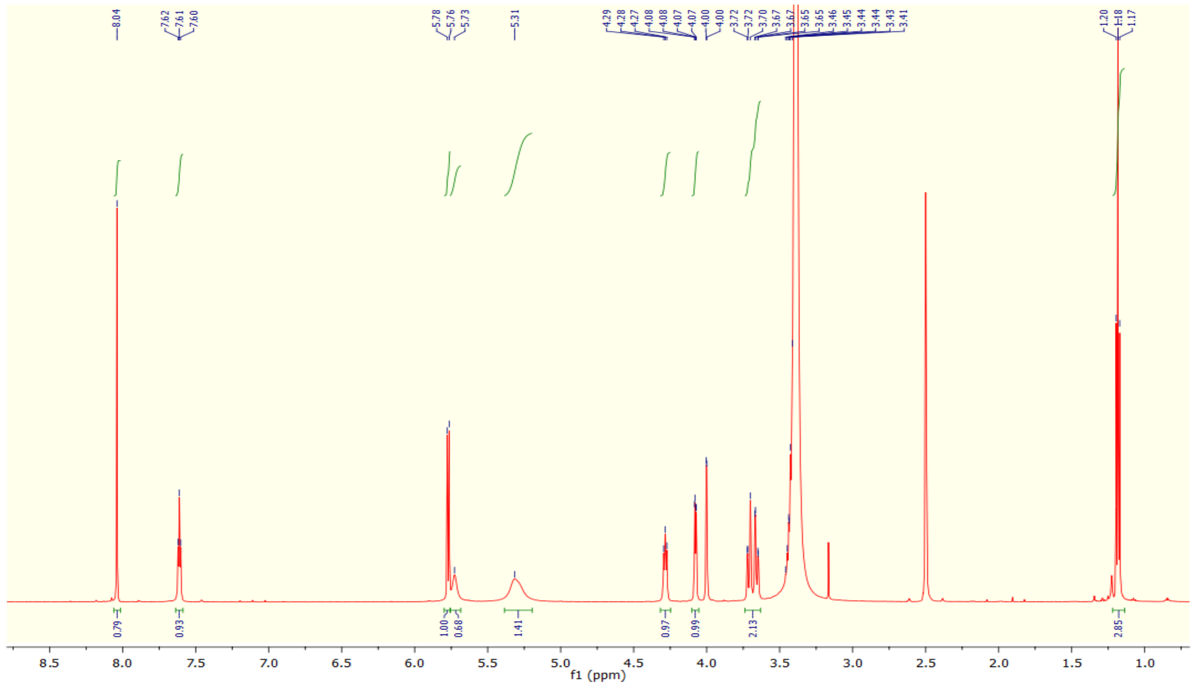

^{13}C-NMR

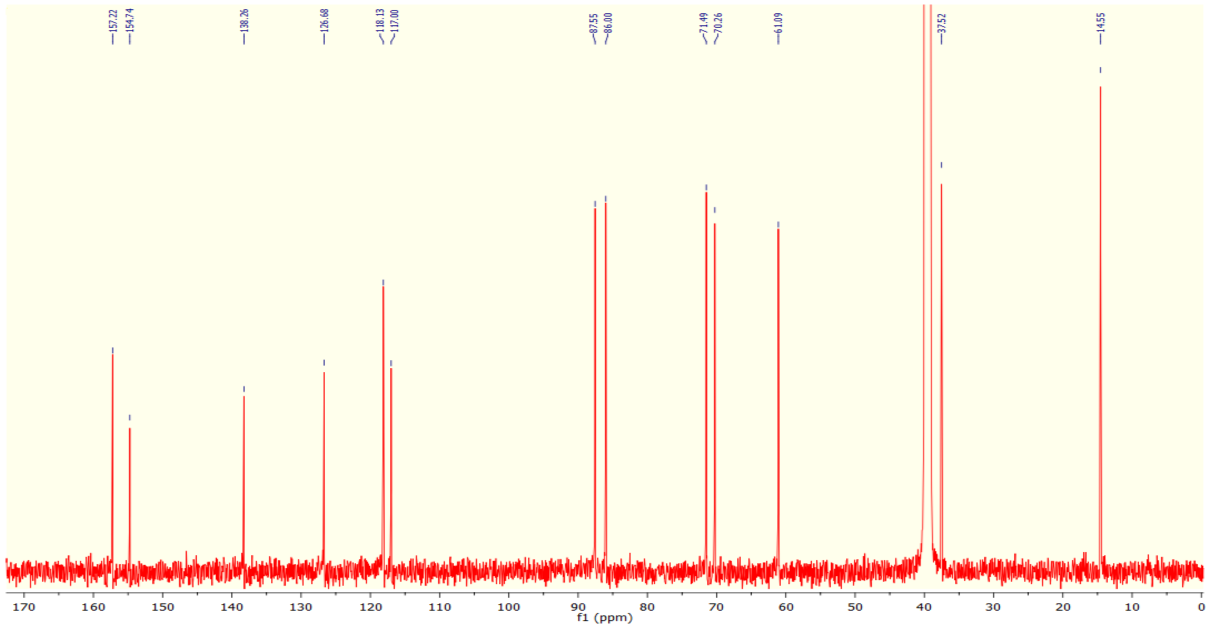

Figure S6: ^{1}H- and ^{13}C-NMR spectra of compound **13a**.

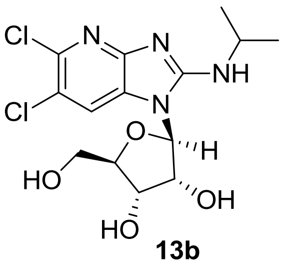

13b

<u>^{1}H-NMR</u>

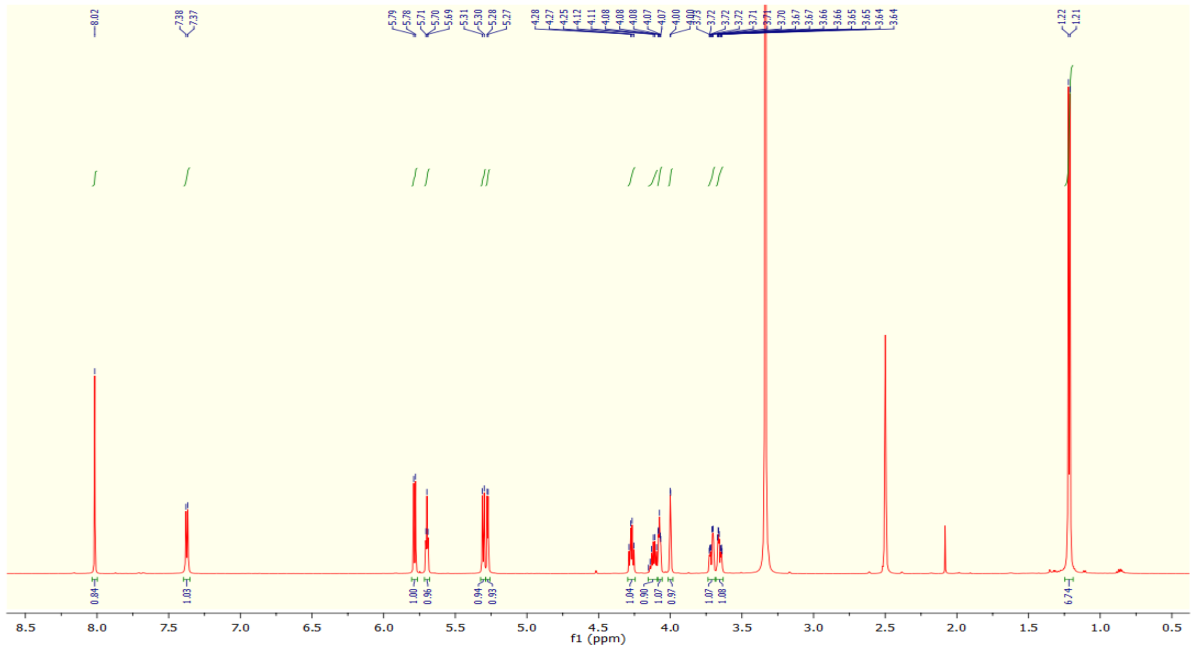

<u>^{13}C-NMR</u>

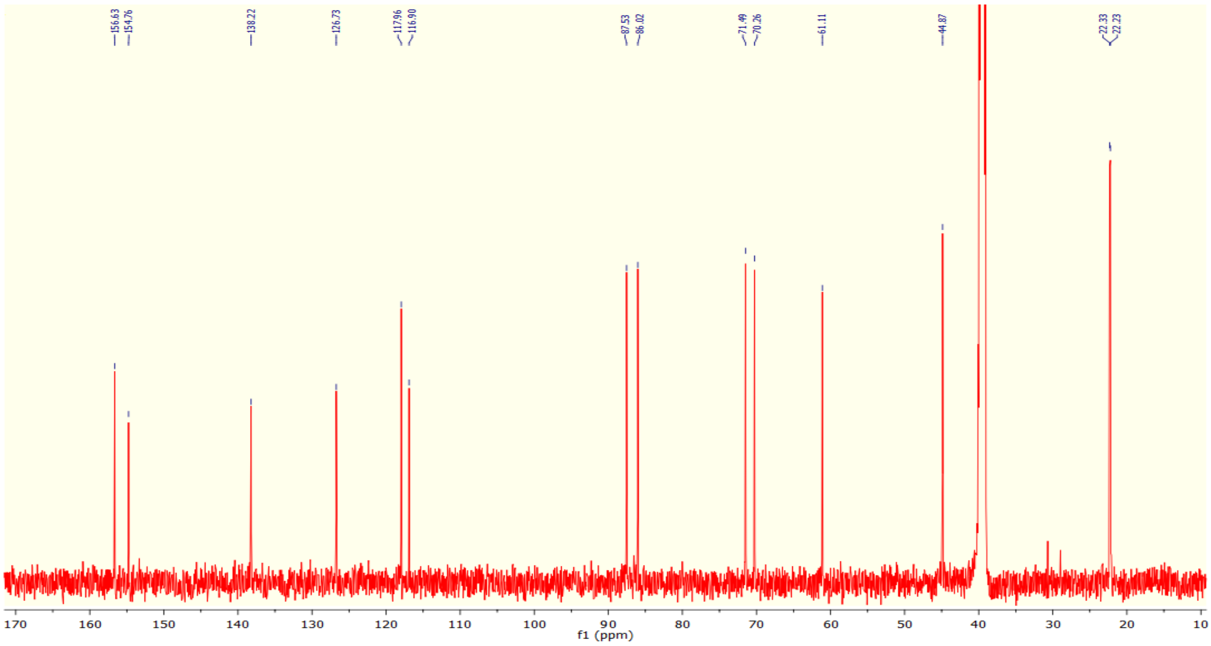

Figure S7: ^{1}H- and ^{13}C-NMR spectra of compound **13b**.

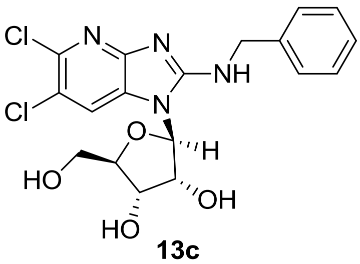

13c

^{1}H-NMR

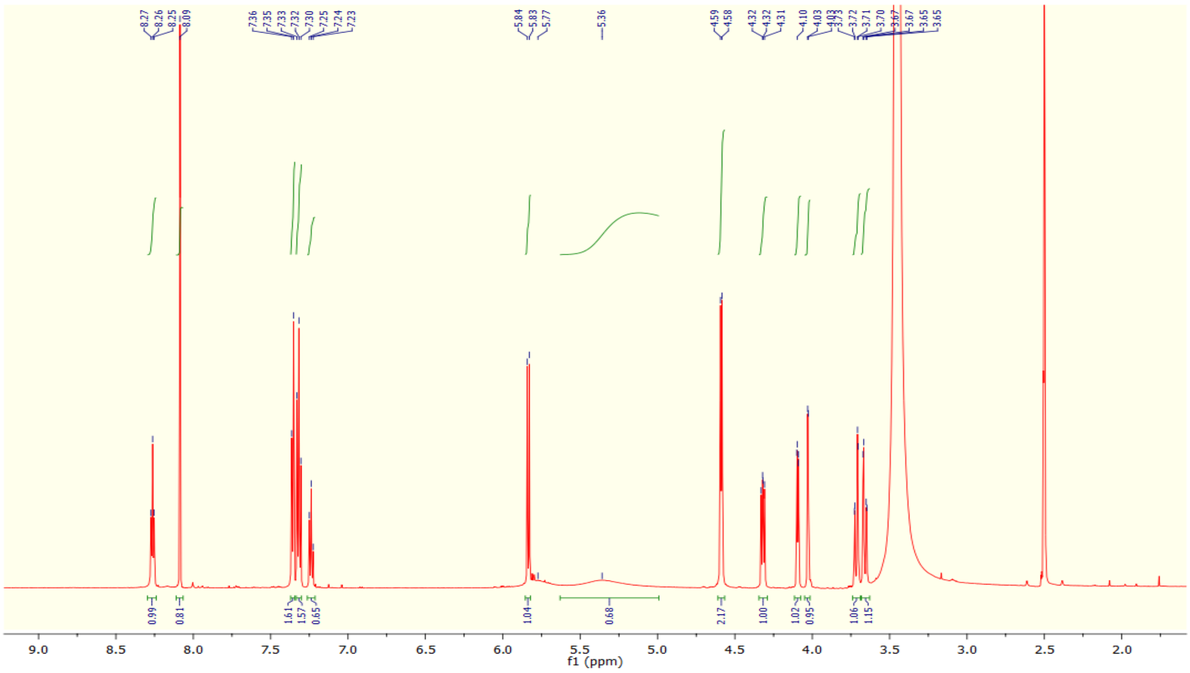

^{13}C-NMR

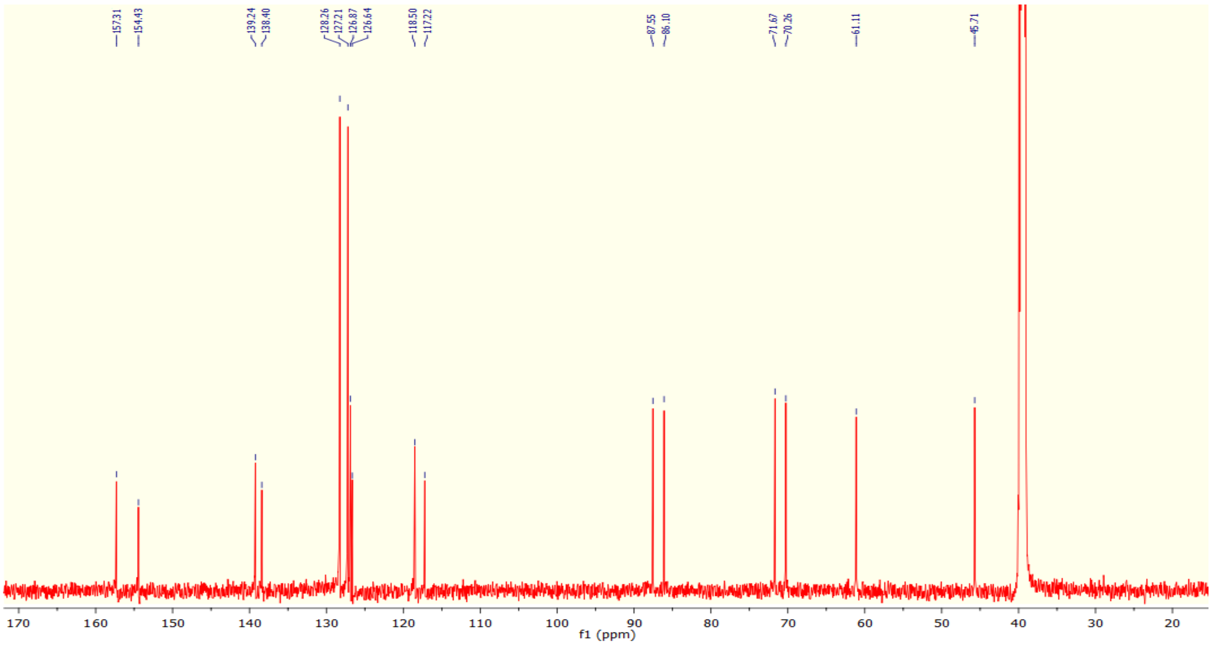

Figure S8: ^{1}H- and ^{13}C-NMR spectra of compound **13c**.

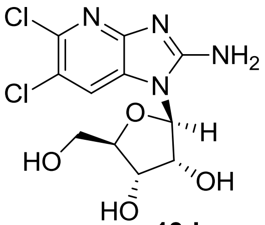

13d

^{1}H-NMR

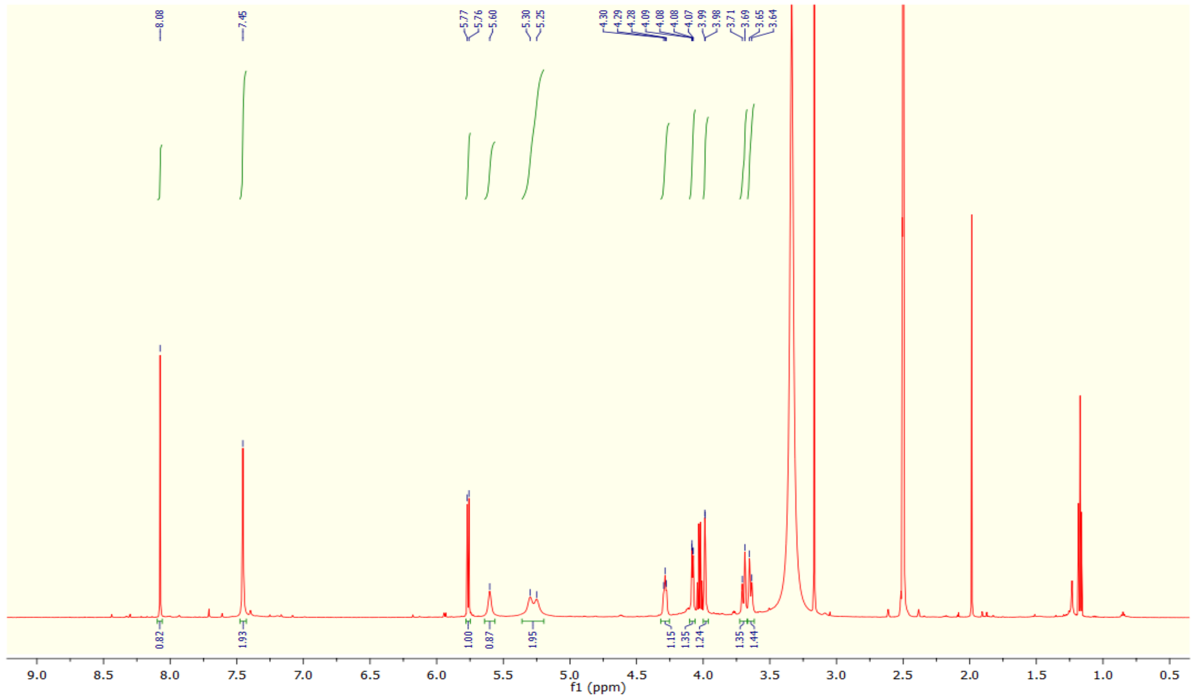

^{13}C-NMR

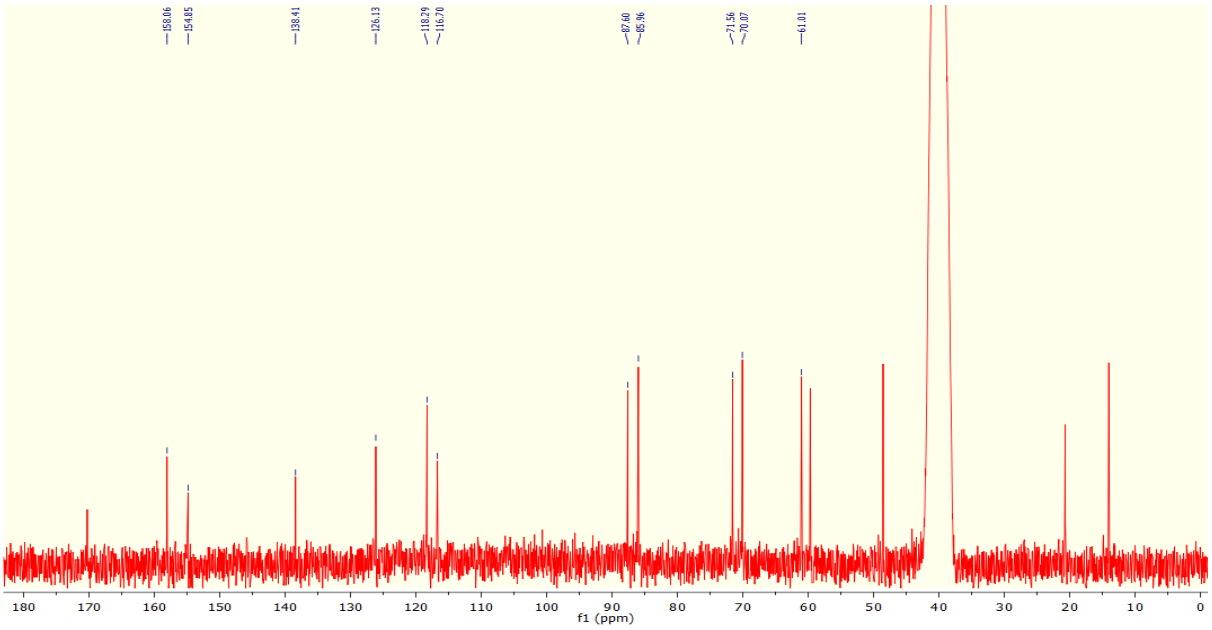

Figure S9: ^{1}H- and ^{13}C-NMR spectra of compound **13d**.

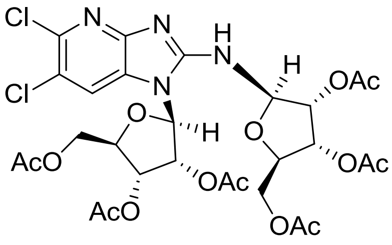

14

^{1}H-NMR

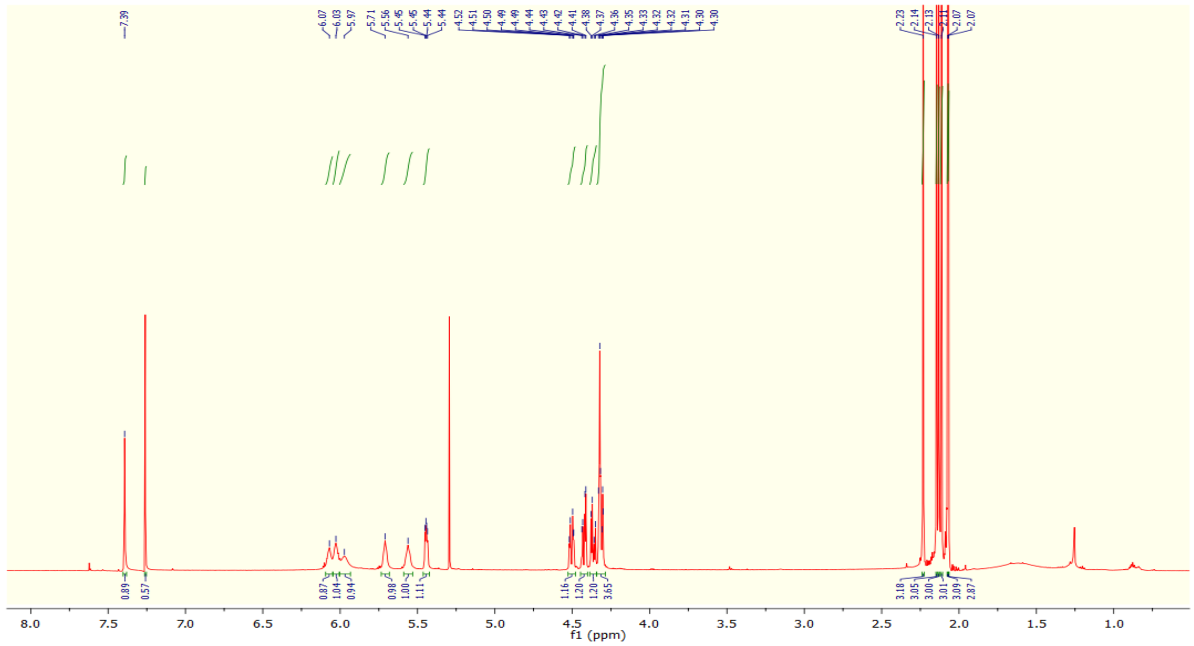

^{13}C-NMR

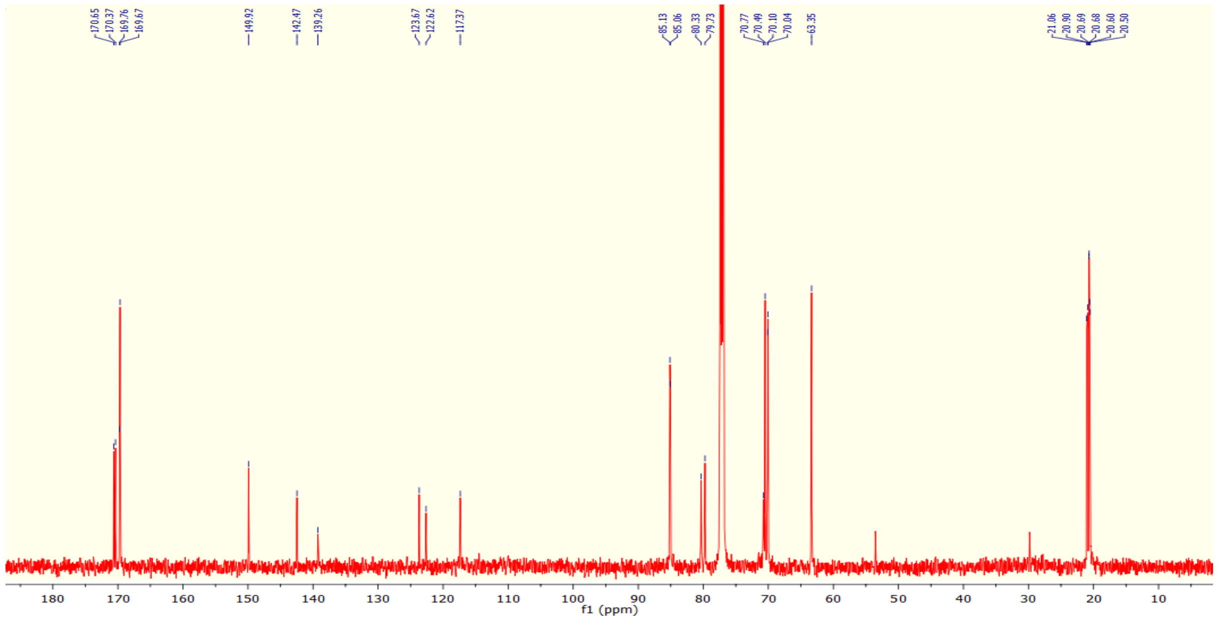

Figure S10: ^{1}H- and ^{13}C-NMR spectra of compound **14**.

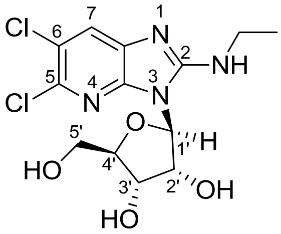

12a

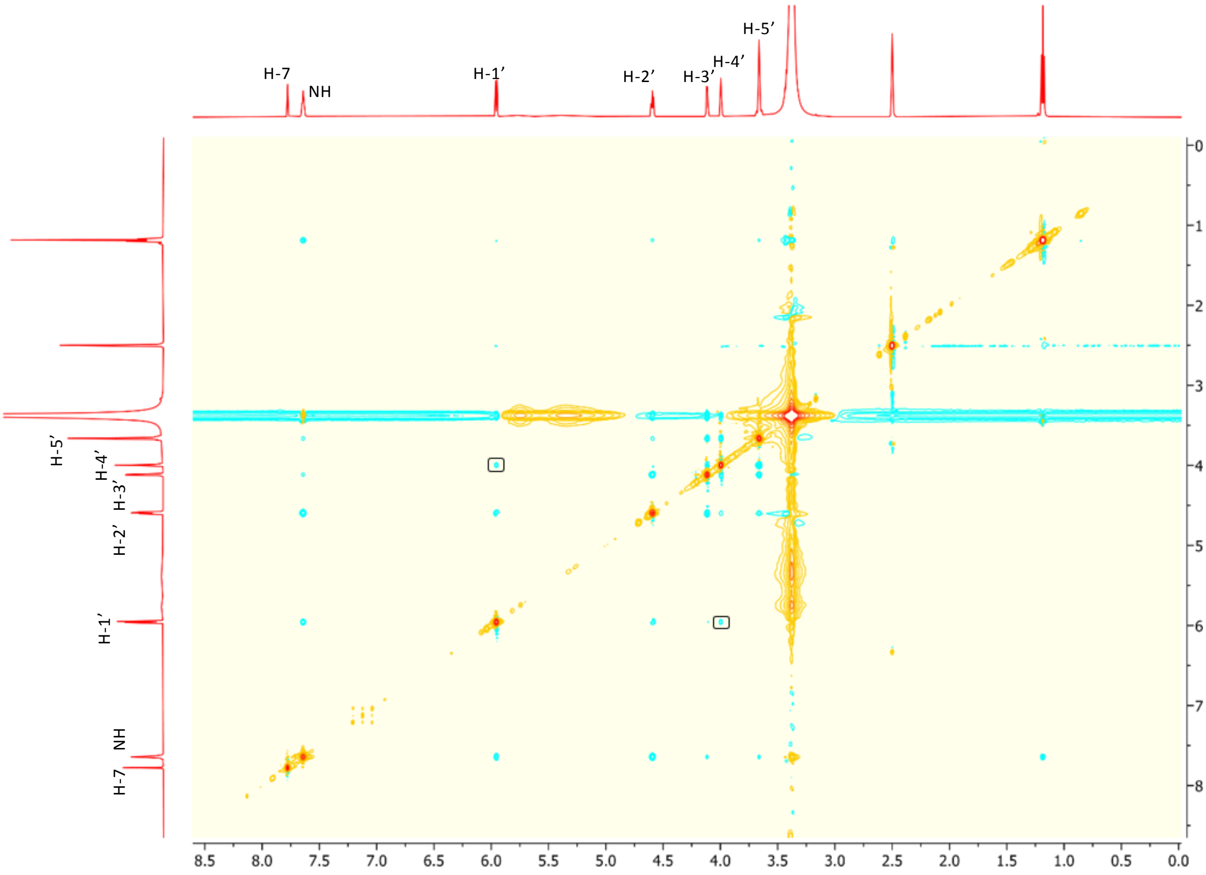

Figure S11: NOE spectrum of compound **12a**. Black circles point out the cross correlation peaks between the H-1’and the H-4’, indicating the *β*- configuration. No correlation peaks of the aromatic proton (H-7) and the protons H-1’, H-2’, H-3’ and H-5’ of the ribofuranosyl moiety are observed.

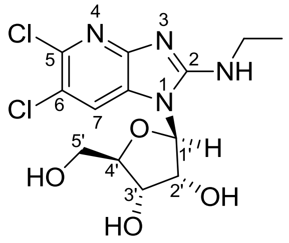

13a

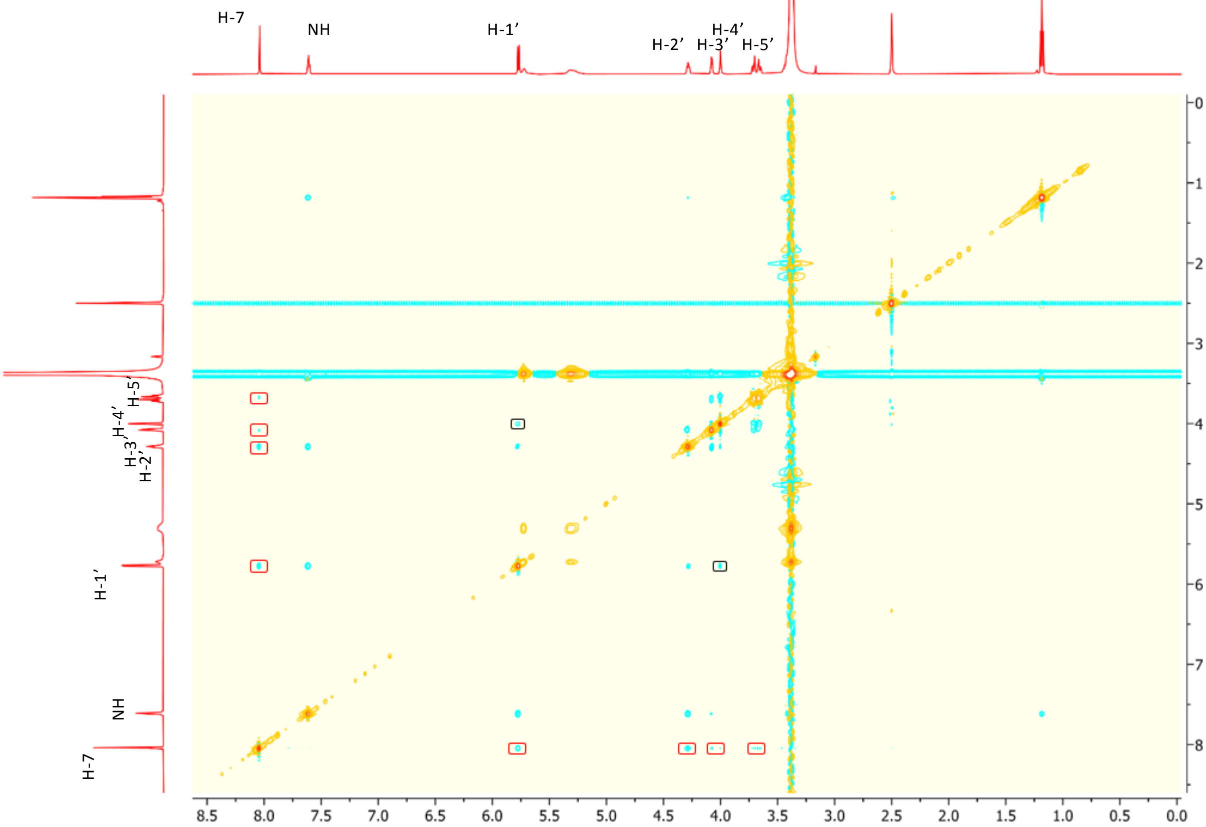

Figure S12: NOE spectrum of compound **13a**. Black circles point out the cross correlation peaks between the H-1’and the H-4’, indicating the *β*- configuration. Correlation peaks of the aromatic proton (H-7) and the protons H-1’, H-2’, H-3’ and H-5’ of the ribofuranosyl moiety are pointed out with red circles, indicating the site of the ribosylation of the imidazopyridine scaffold.

Table S1. Cytotoxicity and antiviral activity of the target compounds against HSV-1, HSV-2, Vaccinia virus, Adeno virus-2 and Human Coronavirus (229E), in HEL cell cultures.

			EC_{50}[b]					
**Compound**	Concentration unit	Minimum cytotoxic concentration[a]	**Herpes simplex virus-1 (KOS)**	**Herpes simplex virus-2 (G)**	**Herpes simplex virus-1 TK⁻ KOS ACV[r]**	**Vaccinia virus**	**Adeno virus-2**	**Human Coronavirus (229E)**
**12a**	µM	>100	>100	>100	>100	>100	>100	>100
**12b**	µM	>100	>100	>100	>100	>100	>100	>100
**12c**	µM	>100	>100	>100	>100	>100	>100	>100
**12d**	µM	>100	>100	>100	>100	>100	>100	>100
**13a**	µM	>100	>100	>100	>100	>100	>100	>100
**13b**	µM	>100	>100	>100	>100	>100	>100	>100
**13c**	µM	>100	>100	>100	>100	>100	>100	>100
**13d**	µM	>100	>100	>100	>100	>100	>100	>100
**Brivudin[c]**	µM	>250	0,04	75	0,5	5,8	-	-
**Cidofovir[c]**	µM	>250	1	2	1	50	10	-
**Acyclovir[c]**	µM	>250	0,4	0,3	3,5	>250	-	-
**Ganciclovir[c]**	µM	>100	0,01	0,03	0,6	>100	-	-
**Zalcitabine[c]**	µM	>250	-	-	-	-	17	-
**Alovudine[c]**	µM	>250	-	-	-	-	5,9	-
**UDA[c]**	µg/ml	>100	-	-	-	-	-	1,8

[a]Required to cause a microscopically detectable alteration of normal cell morphology.
[b]Required to reduce virus-induced cytopathogenicity by 50 %.
[c] Antiviral drugs included as positive controls.

Table S2. Cytotoxicity and antiviral activity of the target compounds against varicella-zoster virus (VZV), in HEL cell cultures.

	**Antiviral activity EC_{50} (µM)[a]**		**Cytotoxicity (µM)**
**Compound**	**TK⁺ VZV strain**	**TK⁻ VZV strain**	**Cell morphology (MCC)[b]**
	OKA	07-1	
**12a**	>100	>100	>100
**12b**	>100	>100	>100
**12c**	>100	>20	20
**12d**	>100	40,27	>100
**13a**	>100	>100	>100
**13b**	>100	>100	>100
**13c**	>100	>20	20
**13d**	>100	>100	>100
**Acyclovir[c]**	2,31	49,06	>440
**Brivudin[c]**	0,0075	0,48	>300

[a] Effective concentration required to reduce virus plaque formation by 50%. Virus input was 20 plaque forming units (PFU).
[b] Minimum cytotoxic concentration that causes a microscopically detectable alteration of cell morphology.
[c]Antiviral drugs included as positive controls.

Table S3. Cytotoxicity and antiviral activity of the target compounds against human cytomegalovirus (HCMV), in HEL cell cultures.

	Antiviral activity EC_{50} (µM)[a]		Cytotoxicity (µM)
**Compound**	**AD-169 strain**	**Davis strain**	**Cell morphology (MCC)[b]**
**12a**	>100	>100	>100
**12b**	>100	>100	>100
**12c**	>20	>4	>100
**12d**	>20	>100	>100
**13a**	>100	>100	>100
**13b**	>100	>100	>100
**13c**	>20	>20	>100
**13d**	>100	>100	>100
**Ganciclovir[c]**	9,22	2,29	>350
**Cidofovir[c]**	1,93	1,05	>300

[a]Effective concentration required to reduce virus plaque formation by 50%. Virus input was 100 plaque forming units (PFU).

[b]Minimum cytotoxic concentration that causes a microscopically detectable alteration of cell morphology.

[c]Antiviral drugs included as positive controls.

Table S4. Inhibitory effects of the target compounds on the proliferation of human T-lymphocyte cells (CEM), human cervix carcinoma cells (HeLa) and human dermal microvascular endothelial cells (HMEC-1).

	IC_{50} (µM)[a]		
**Compound**	**CEM**	**HeLa**	**HMEC-1**
**12a**	>100	>100	>100
**12b**	>100	>100	>100
**12c**	37 ± 3	36 ± 7	20 ± 2
**12d**	49 ± 9	40 ± 1	57 ± 8
**13a**	>100	>100	>100
**13b**	>100	>100	>100
**13c**	39 ± 8	≥ 100	50 ± 4
**13d**	>100	>100	>100

[a] Concentration of the compound necessary for 50% of growth inhibition. The IC_{50} values were calculated from concentration-response curve using linear regression analysis. Each test was performed in quadruplicate in at least two individual experiments.
